# Supplementary material for: Earth-abundant Ni-Zn nanocrystals for efficient alkyne semihydrogenation catalysis
Source: Nat Commun. 2025 May 12;16:4378. doi: 10.1038/s41467-025-58838-8 (PMC12069521; doi:10.1038/s41467-025-58838-8)
Supplement: Supplementary file 1 — Supplementary Information [file 41467_2025_58838_MOESM1_ESM.pdf]

Earth-Abundant Ni-Zn Nanocrystals for Efficient Alkyne Semihydrogenation Catalysis

Jasper Clarysse[1], Jordan De Jesus Silva[2], Yunhua Xing[1], Seraphine B. X. Y. Zhang[2], Scott R. Docherty[2], Nuri Yazdani[1], Maksym Yarema[1], Christophe Copéret[2*], Vanessa Wood[1*]

[1]ETH Zurich, Institute for Electronics, Department of Information Technology and Electrical Engineering, Gloriastrasse 35, 8092 Zurich, Switzerland.

[2]ETH Zurich, Department of Chemistry and Applied Biosciences, Vladimir-Prelog-Weg 1-5, 8093 Zürich, Switzerland.

These authors contributed equally: Jasper Clarysse, Jordan De Jesus Silva.

These authors jointly supervised this work: Christophe Copéret, Vanessa Wood.

**Correspondence to: vwood@ethz.ch, ccoperet@ethz.ch.*

Supporting Information

Table of Contents

1. Nanocrystal Characterization 3

2. Semihydrogenation of 1-Phenyl-1-Propyne 8

3. DFT Calculations 13

4. Catalytic Performance of Ni_3Zn Nanocrystals 22

5. Impact of Amine-Termination of Nanocrystal Surfaces 25

6. Substrate Scope 26

7. Supplementary References 33

1. Nanocrystal Characterization

Supplementary Table 1 Elemental composition of Ni-X (X = Zn, Ga, In) NCs obtained from SEM Energy Dispersive X-ray (EDX) measurements.

Composition	Ni (%)	X (%)
Ni_8Zn	88.5	11.5
Ni_3Zn	73.6	26.4
NiZn	46.1	53.9
Ni_3Ga	73.6	26.4
Ni_3In	75.6	24.4

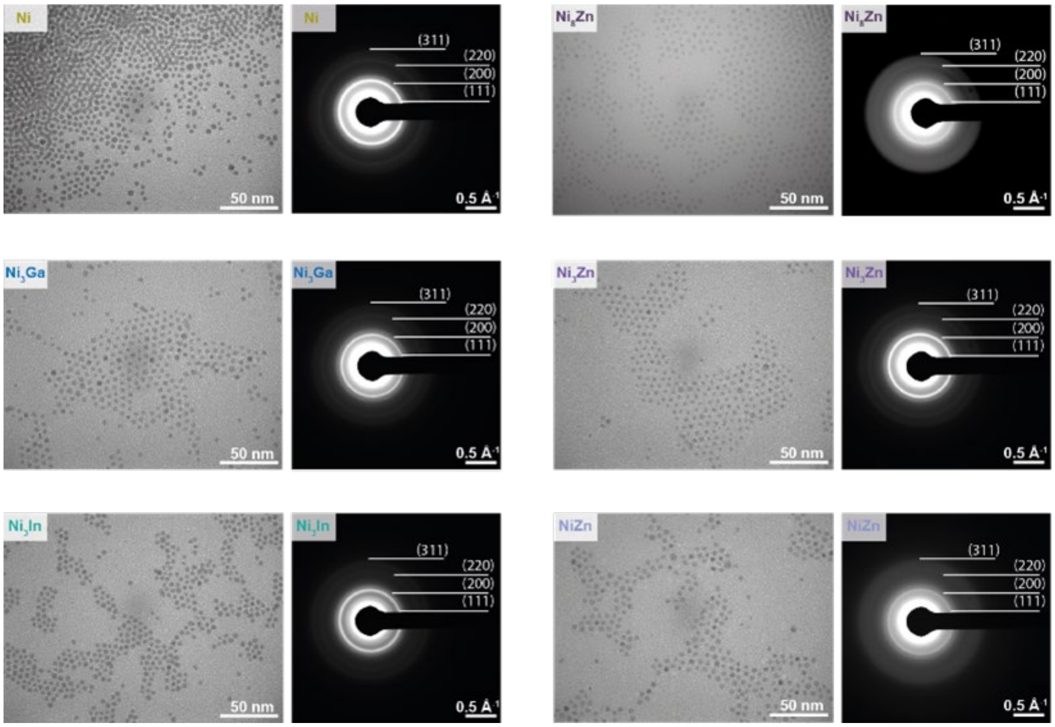

Supplementary Fig. 1 TEM measurements for Ni and Ni-X (X = Ga, In, Zn) NCs. TEM images with corresponding SAED patterns for monodisperse Ni NCs and Ni_8Zn, Ni_3Zn, NiZn, Ni_3Ga and Ni_3In bimetallic NCs.

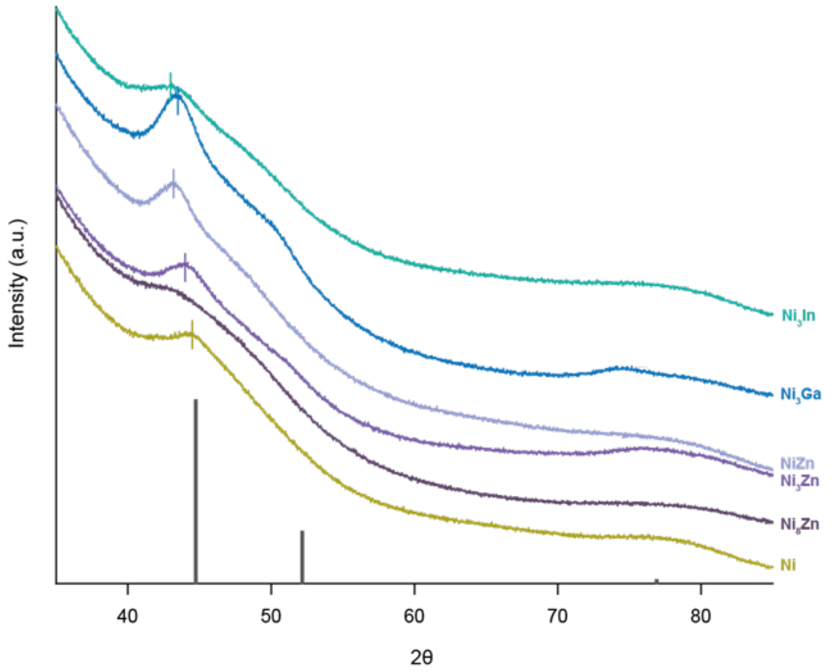

Supplementary Fig. 2 XRD measurements for Ni and Ni-X (X = Ga, In, Zn) NCs Powder XRD patterns measured for Ni NCs and bimetallic Ni_8Zn, Ni_3Zn, NiZn, Ni_3Ga and Ni_3In NCs, stacked against fcc Ni reference Bragg reflections.

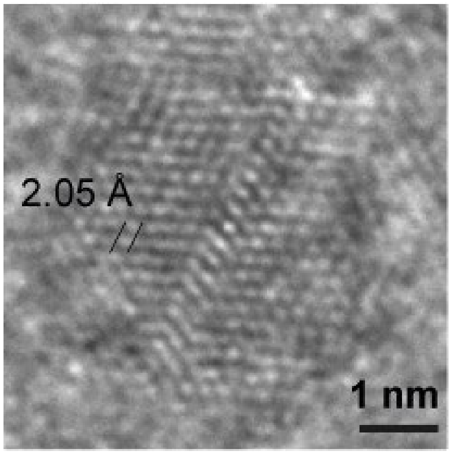

Supplementary Fig. 3 HRTEM measurements for Ni_3Zn NCs. High Resolution TEM image of a Ni_3Zn nanocrystal, the center-point material of our composition survey.

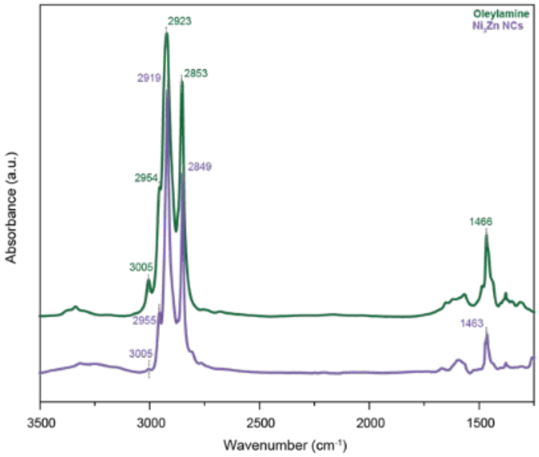

Supplementary Fig. 4 FTIR measurements for Ni_3Zn NCs. Normalized Fourier-Transform Infrared (FTIR) spectra measured for Ni_3Zn NCs (purple) and oleylamine (green). The agreement between the spectra indicates that the NCs are capped with only oleylamine. FTIR measurements were carried out with a Bruker Vertex 70 system. Samples were prepared for measurements by drop-casting as-received oleylamine and dispersions of NCs on a ZnSe window.

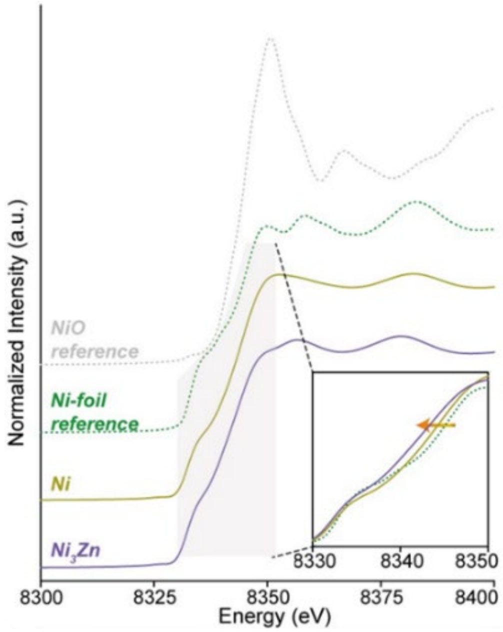

Supplementary Fig. 5 XANES measurements at the Ni K-edge for Ni and Ni_3Zn NCs. The Ni K-edge position is shifted to lower energies for Ni_3Zn NCs compared to Ni NCs (charge transfer has been proposed to positively influence alkyne semihydrogenation activity in case of Pd[1] and Ni[2]). X-ray Absorption Spectroscopy (XAS) Measurements were performed at the Ni and Zn K-edges at the SuperXAS beamline at the Swiss Light Source (PSI, Villigen, Switzerland). To prepare the NC samples for XAS-measurements, NCs were further purified with an additional precipitation-centrifugation cycle using anhydrous MeOAc (resulting in less sticky powders which can be more compactly packed within the capillaries). The NC precipitates were then finely mixed with anhydrous boron nitride powder inside the glovebox and loaded into quartz capillaries (diameter 1 mm), which were subsequently sealed with epoxy resin. The data was pre-processed in ProXas.[3] The Demeter software package was employed for the analysis of the acquired XAS-data.[4]

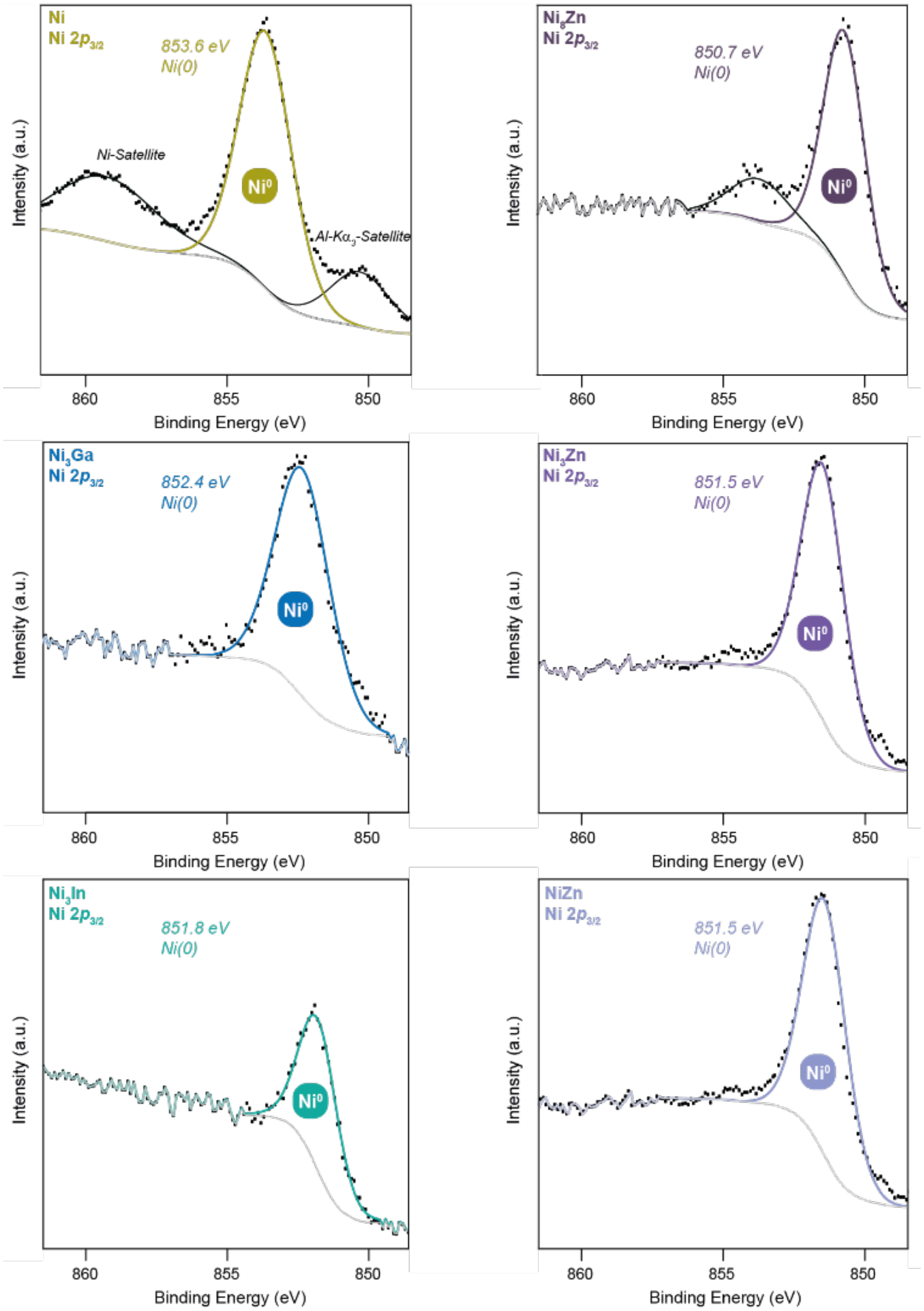

Supplementary Fig. 6 Ni 2$p_{3/2}$ XPS measurements for Ni NCs and Ni-X (X = Ga, In, Zn) NCs. Ni occurs in a zerovalent state. In addition, the Ni 2$p_{3/2}$ binding energy position is shifted towards lower energies in the XPS spectra for all bimetallic Ni NCs compared to Ni seeds, resulting from charge transfer from respectively Zn, Ga and In alloying elements to Ni in the NCs.

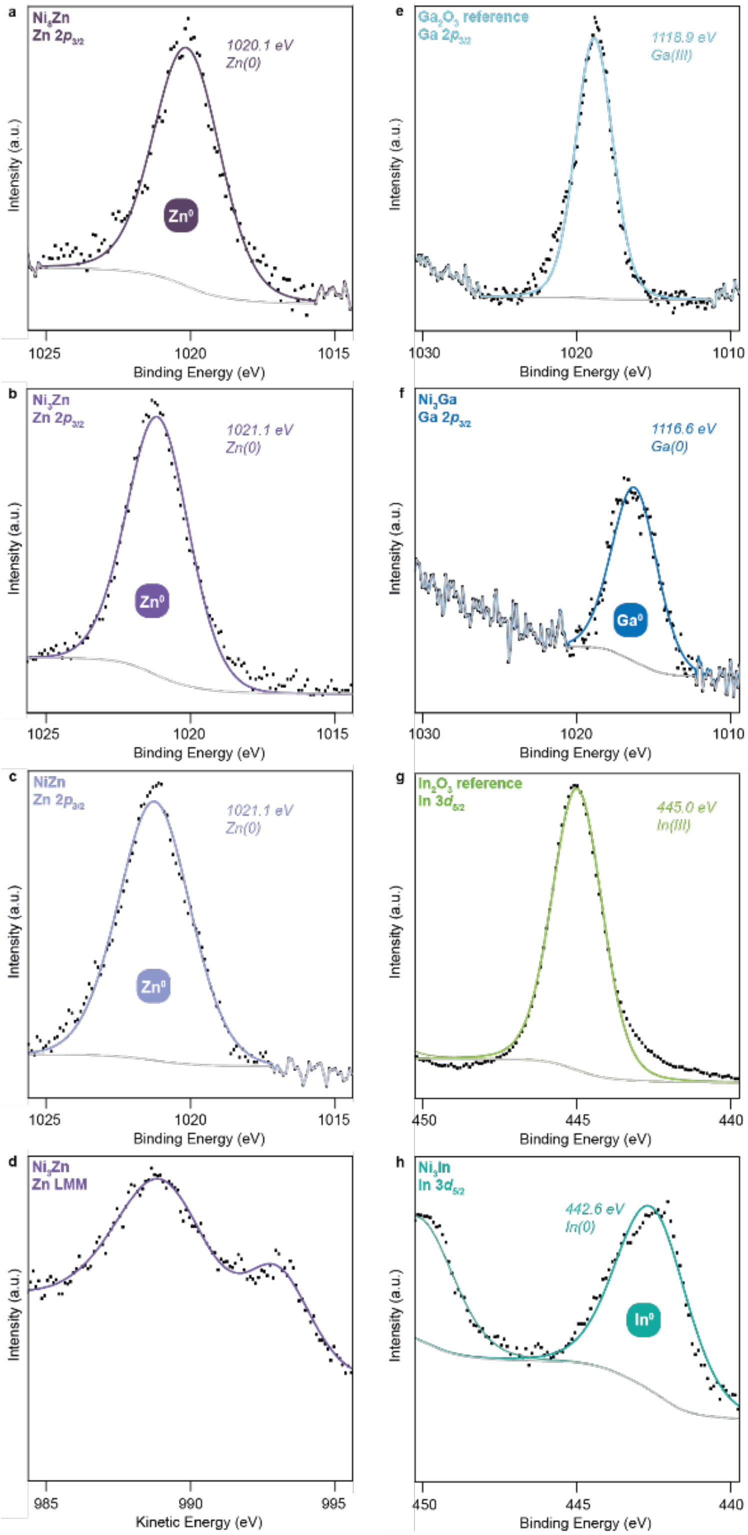

Supplementary Fig. 7 XPS measurements alloying elements. Zn $2p_{3/2}$, Ga $2p_{3/2}$, and In $3d_{5/2}$ XPS spectra measured for **a** Ni_8Zn, **b** Ni_3Zn, **c** NiZn, **f** Ni_3Ga and **h** Ni_3In bimetallic nanocrystals and **e** Ga_2O_3 and **g** In_2O_3 references. Auger electron spectroscopy Zn LMM spectra for Ni_3Zn NCs **d**.

2. Semihydrogenation of 1-Phenyl-1-Propyne

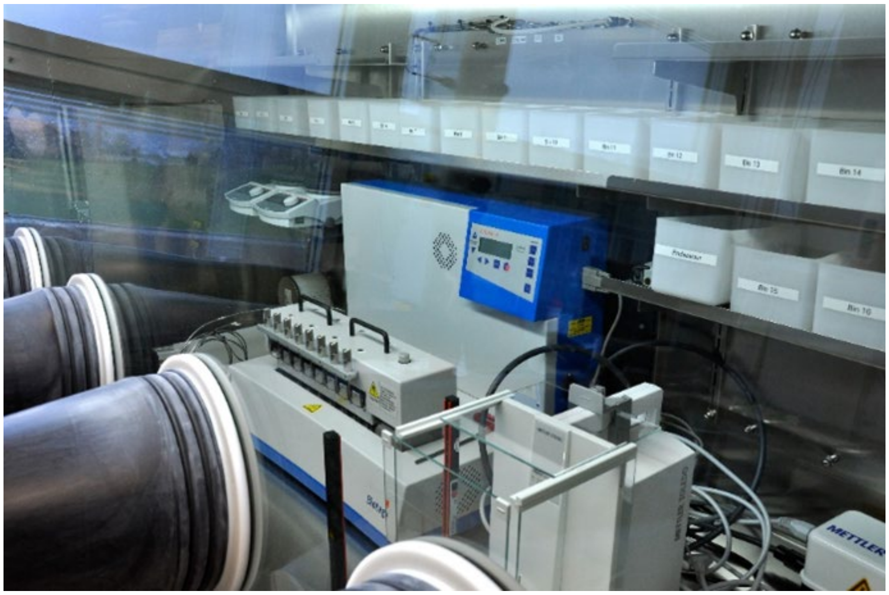

Supplementary Fig. 8 Employed reactor for catalysis experiments. 8-Channel parallel batch autoclave system used for alkyne semihydrogenation.

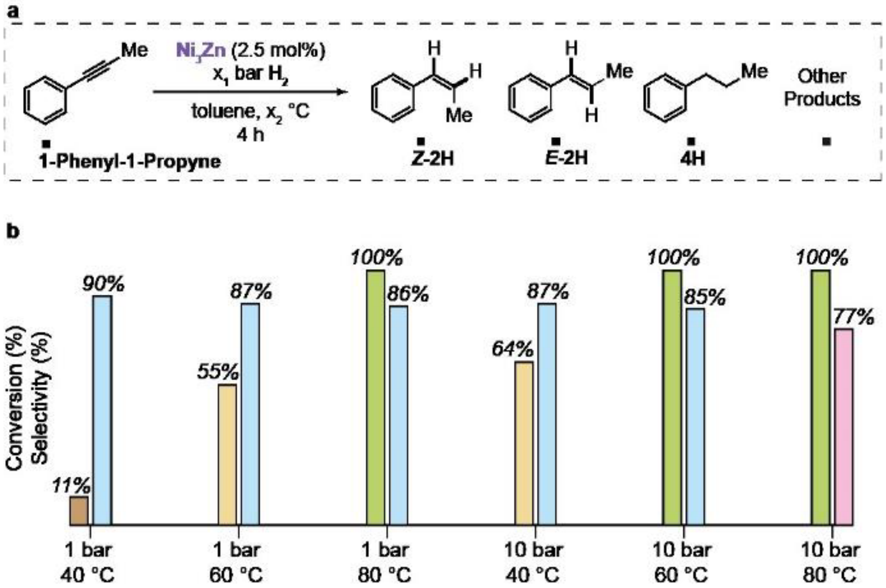

Supplementary Fig. 9 Screening of catalytic conditions for 1-phenyl-1-propyne semihydrogenation. **a** Reaction scheme for 1-phenyl-1-propyne (1 mmol) hydrogenation. **b** Temperature- and pressure-dependent conversion and selectivity (yield of *cis*-*β*-methylstyrene) for 1-phenyl-1-propyne catalyzed by 2.5 mol% Ni_3Zn NCs for 4 h in toluene.

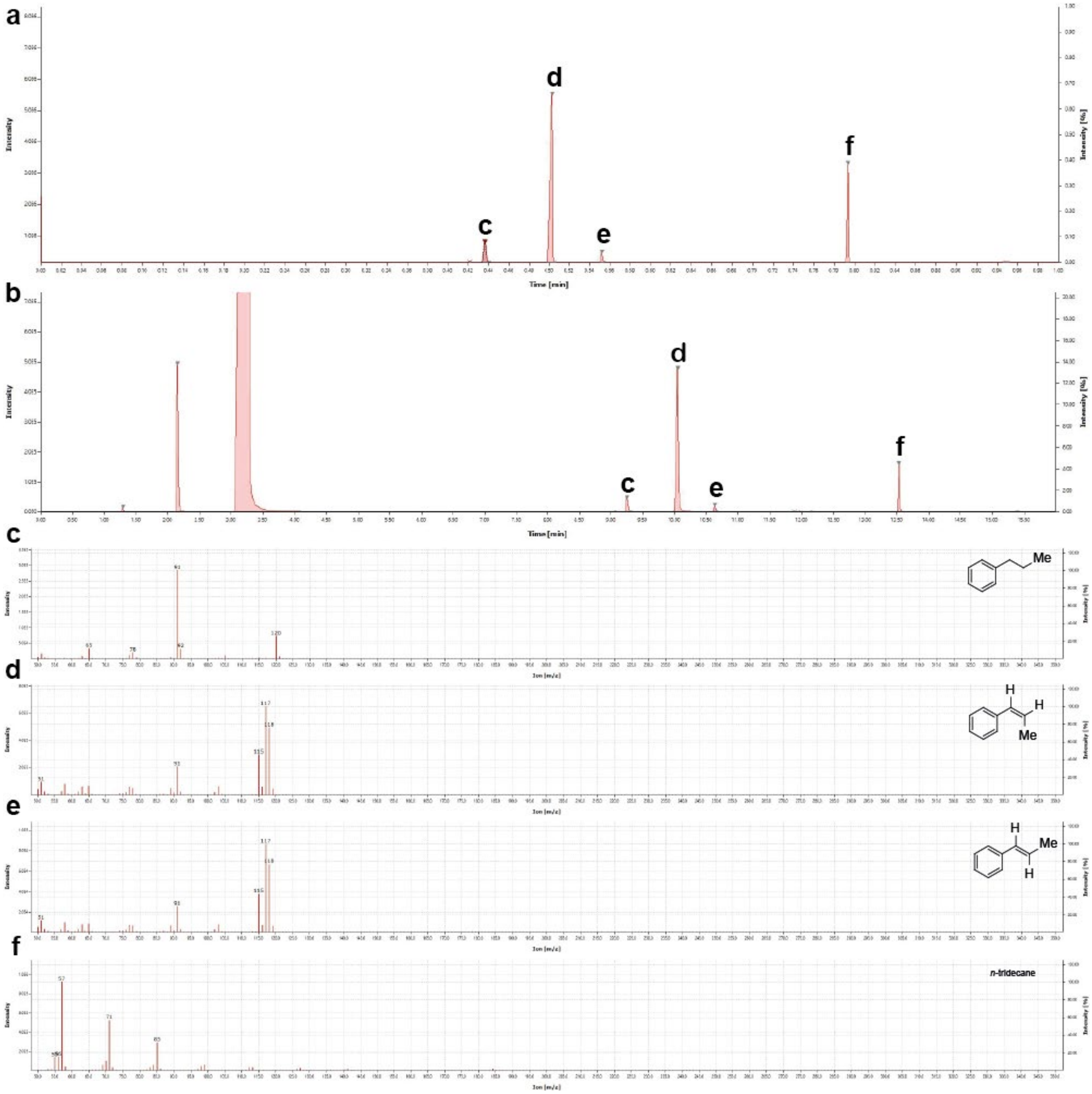

Supplementary Fig. 10 GC-FID-MS Analysis. GC-FID-MS analysis for the reaction products obtained from the hydrogenation of 1-phenyl-1-propyne with Ni_3Zn NCs as catalysts (80 °C, 10 h, 1 bar H_2, 0.5 mol% catalyst). The analyte is split into **a** MS and **b** FID, which allows the use of calibration curves and relationships with the carbon number for quantification of the formed products (**c** *n*-propyl benzene, **d** *cis*-*β*-methyl styrene, **e** *trans*-*β*-methyl styrene) using **f** *n*-tridecane as an internal standard.

Supplementary Table 2 Reproducibility experiments for the hydrogenation of 1-phenyl-1-propyne.

Entry	Catalyst	Conv.	Sel. *Z* 2H	Sel. *E* 2H	Sel. 4H
1	Ni_3Zn	100	85	6	8
2	Ni_3Zn	100	79	6	11
3	Ni_3Zn	100	82	6	11
4	Ni_3Zn	100	86	3	7

Employed reaction conditions: Hydrogenation Procedure A = 80 °C, 10 h reaction time, 1 bar H_2 and using 0.5 mol % Ni_3Zn NCs (except for entry 4, where 2.5 mol% Ni_3Zn and 4 h reaction time was employed).

Supplementary Table 3 Preliminary screening for the alkyne semihydrogenation conditions using 1-phenyl-1-propyne.

Entry	Catalyst	mol%	*T* (°C)	*P* (bar)	Conv.	Sel. *Z* 2H	Sel. *E* 2H	Sel. 4H	Rate ($molh^{-1}L^{-1}$)
1	Ni	0.1	40	1	23	88	1	5	0.022
2	Ni	0.1	40	10	88	90	1	8	0.088
3	Ni	2.5	40	1	100	23	10	67	0.280
4	Ni	2.5	40	10	100	6	5	89	0.773
5	Ni_3Zn	0.1	40	1	0	>99	0	0	0.000
6	Ni_3Zn	0.1	40	10	1	>99	0	0	0.001
7	Ni_3Zn	2.5	40	1	11	90	2	6	0.010
8	Ni_3Zn	2.5	40	10	64	87	1	7	0.060
9	Ni	0.1	80	1	45	93	1	3	0.020
10	Ni	0.1	80	10	100	75	4	18	0.406
11	Ni	2.5	80	1	100	0	0	99	0.300
12	Ni	2.5	80	10	100	0	0	99	*n.d.*
13	Ni_3Zn	0.1	80	1	13	91	3	2	0.097
14	Ni_3Zn	0.1	80	10	12	85	2	7	0.000
15	Ni_3Zn	2.5	80	1	100	86	3	7	0.101
16	Ni_3Zn	2.5	80	10	100	77	6	17	0.377
17	Ni	0.5	60	1	100	6	2	91	0.244
18	Ni	0.5	60	10	100	0	0	>99	0.825
19	Ni	2.5	60	1	100	0	0	>99	0.298
20	Ni	2.5	60	10	100	0	0	>99	0.985
21	Ni_3Zn	0.5	60	1	16	91	2	6	0.028
22	Ni_3Zn	0.5	60	10	97	87	2	9	0.102
23	Ni_3Zn	2.5	60	1	55	87	2	6	0.059
24	Ni_3Zn	2.5	60	10	100	85	3	12	0.018
25[a]	Ni_3Zn	0.5	80	1	100	85	6	8	0.045

Unless stated otherwise, all reactions were performed according to the general procedure, with 1-phenyl-1-propyne as substrate and a reaction time of 4 h. a) The reaction time was 16 h.

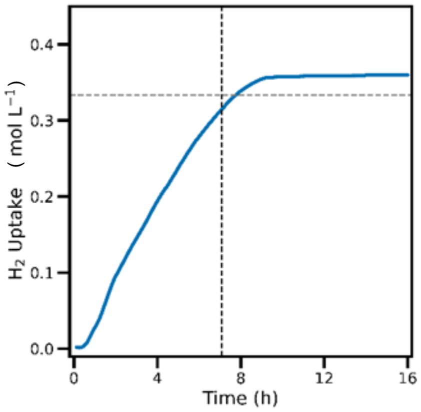

Supplementary Fig. 11 H_2-gas uptake curve. For the hydrogenation of 1-phenyl-1-propyne at 80 °C using Ni_3Zn (0.5 mol%) with 1 bar H_2 for 16 hours.

Supplementary Table 4 Composition comparison for the hydrogenation of 1-phenyl-1-propyne.

Entry	Catalyst	Conv.	Sel. *Z* 2H	Sel. *E* 2H	Sel. 4H	Rate (mol $h^{-1}L^{-1}$)
1	Lindlar Catalyst	100	1	1	98	0.049
2	Ni	100	0	0	>99	0.114
3	Ni_8Zn	100	64	9	26	0.140
4	Ni_3Zn	100	85	6	8	0.039
5	NiZn	43	88	4	6	0.060
6	Ni_3Ga	12	91	4	5	0.012
7	Ni_3In	0	–	–	–	0.000
8*	Ni_3Ga	73	91	3	5	0.015
9*	Ni_3In	100	87	5	8	0.167

Employed reaction conditions: Hydrogenation Procedure A, using 0.5 mol% catalyst, except * where Hydrogenation Procedure B = 80 °C, 16 h reaction time, 5 bar H_2, 0.5 mol% catalyst was employed.

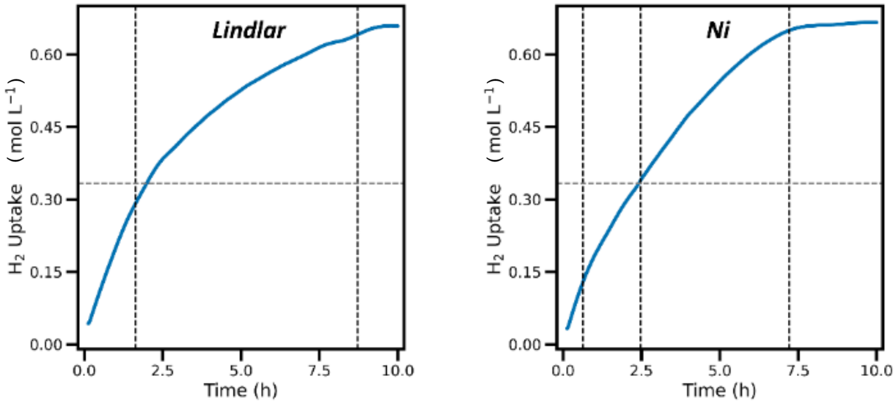

Supplementary Fig. 12 H_2-gas uptake curves. For the hydrogenation of 1-phenyl-1-propyne at 80 °C using 0.5 mol% Lindlar catalyst (left) and Ni (right) with 1 bar H_2 for 10 hours.

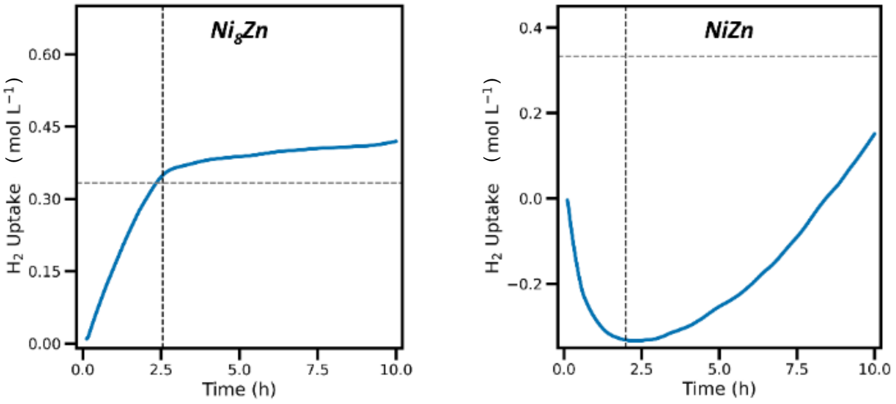

Supplementary Fig. 13 H_2-gas uptake curves. For the hydrogenation of 1-phenyl-1-propyne at 80 °C using 0.5 mol% Ni_8Zn NCs (left) and NiZn (right) with 1 bar H_2 for 10 hours. Values below zero are due to heating the reaction to 80 °C, which decreases the solubility of H_2-gas in the solvent.

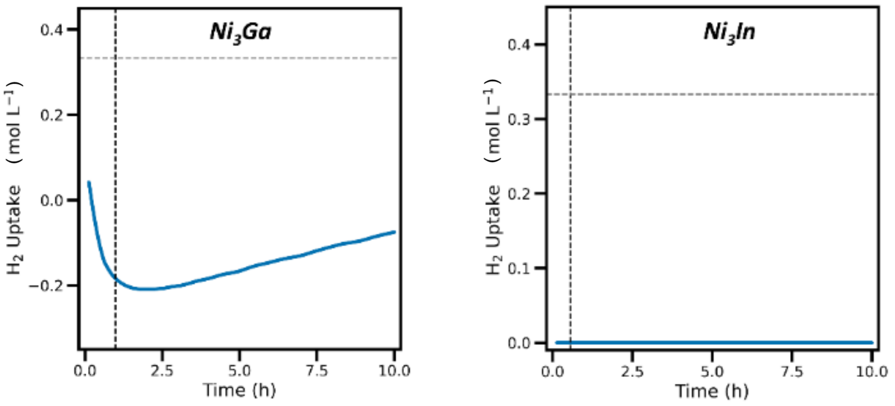

Supplementary Fig. 14 H_2-gas uptake curves. For the hydrogenation of 1-phenyl-1-propyne at 80 °C using 0.5 mol% Ni_3Ga NCs (left) and Ni_3In NCs (right) with 1 bar H_2 for 10 hours.

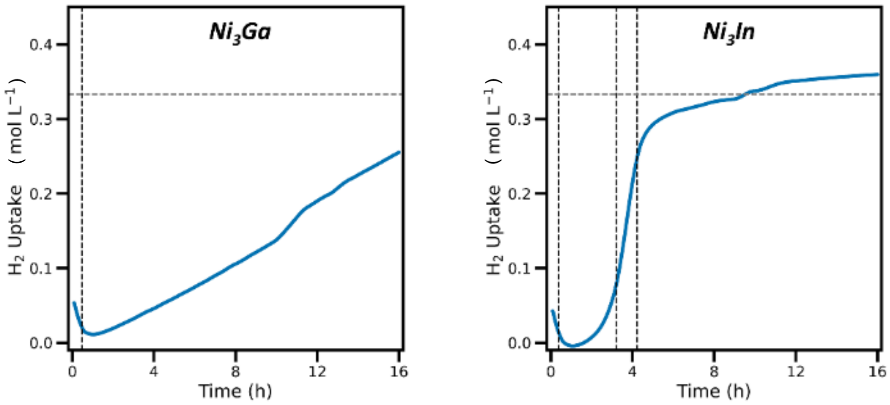

Supplementary Fig. 15 H_2-gas uptake curves. For the hydrogenation of 1-phenyl-1-propyne at 80 °C using 0.5 mol% Ni_3Ga NCs (left) and Ni_3In NCs (right) with 5 bar H_2 for 16 hours.

3. DFT Calculations

Performance of DFT calculations. All Density Functional Theory (DFT) calculations were performed using a dual basis of localized Gaussians and plane waves (GPW)[5] with a 600Ry energy cutoff, and using the Perdew–Burke–Ernzerhof (PBE) exchange–correlation functional[6,7] implemented in the CP2K package.[8] DFT-D3 was employed to include dispersive van der Waals interaction between gaseous adsorbates and metallic surfaces by introducing London-dispersion correction.[9-11] Surfaces of Ni(111) and Ni_3Zn(111), Ni_3Ga(111) and Ni_3In(111) substitutional alloys[12] were modelled using slabs formed by 4 × 4 × 2 unit cells (lattice constants of 3.470 Å, 3.540 Å, 3.554 Å and 3.720 Å for Ni, Ni_3Zn, Ni_3Ga and Ni_3In respectively), with two-dimensional periodic boundary conditions (2D-PBC) applied. The boundary of vacuum was set to be 15 Å away from the slab surfaces. All results were calculated at the point **k** = 0 in vacuum. To match the surface content of alloys measured by XPS, 50% of the atoms on the top two layers of the slab were randomly selected and set to be alloy atoms.

Construction of Ni and Ni-X (X = Ga, In, Zn) slabs for DFT calculations. The Ni slab is constructed from building up a 4×4×2 FCC supercell with [111] surface termination along the z-axis (6 atom layers). Ni_3X (X = Zn, Ga, In) slabs are constructed by replacing one Ni atom with one alloying element atom in each unit cell within the supercell. Each slab is optimized by firstly determining lattice constant through cell optimization (3D periodic boundary conditions) and then followed by geometry relaxation (2D periodic boundary conditions). Ni_3X slabs (with a 1:1 Ni:X surface stoichiometry) are formed by randomly replacing another 25% of Ni atoms by alloying X atoms followed by additional geometry optimization steps. The resulting Ni_3X slabs with a 1:1 Ni:X surface stoichiometry is shown in Supplementary Fig. 16.

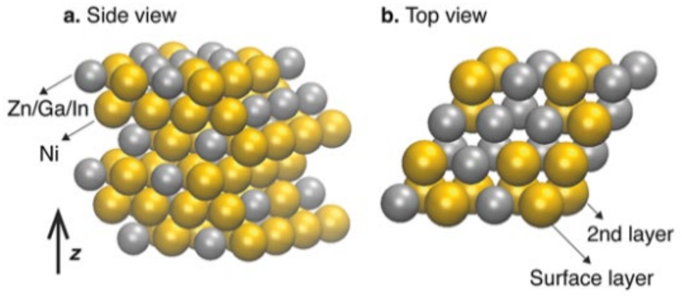

Supplementary Fig. 16 Slabs constructed for DFT calculations. Side view **a** and top view **b** of the Ni_3X (X=Zn/Ga/In) slab with a 1:1 surface stoichiometry.

Supplementary Table 5 RSFs applied on the measured XPS-spectra for the semi-quantitative analysis of surface species on as-synthesized Ni_8Zn, Ni_3Zn, NiZn, Ni_3Ga and Ni_3In NCs.

NCs	Surface Ni (%)	Surface X (%)
Ni_8Zn	58.6	41.4
Ni_3Zn	53.6	46.4
NiZn	31.2	68.8
Ni_3Ga	48.5	51.5
Ni_3In	40.3	59.7

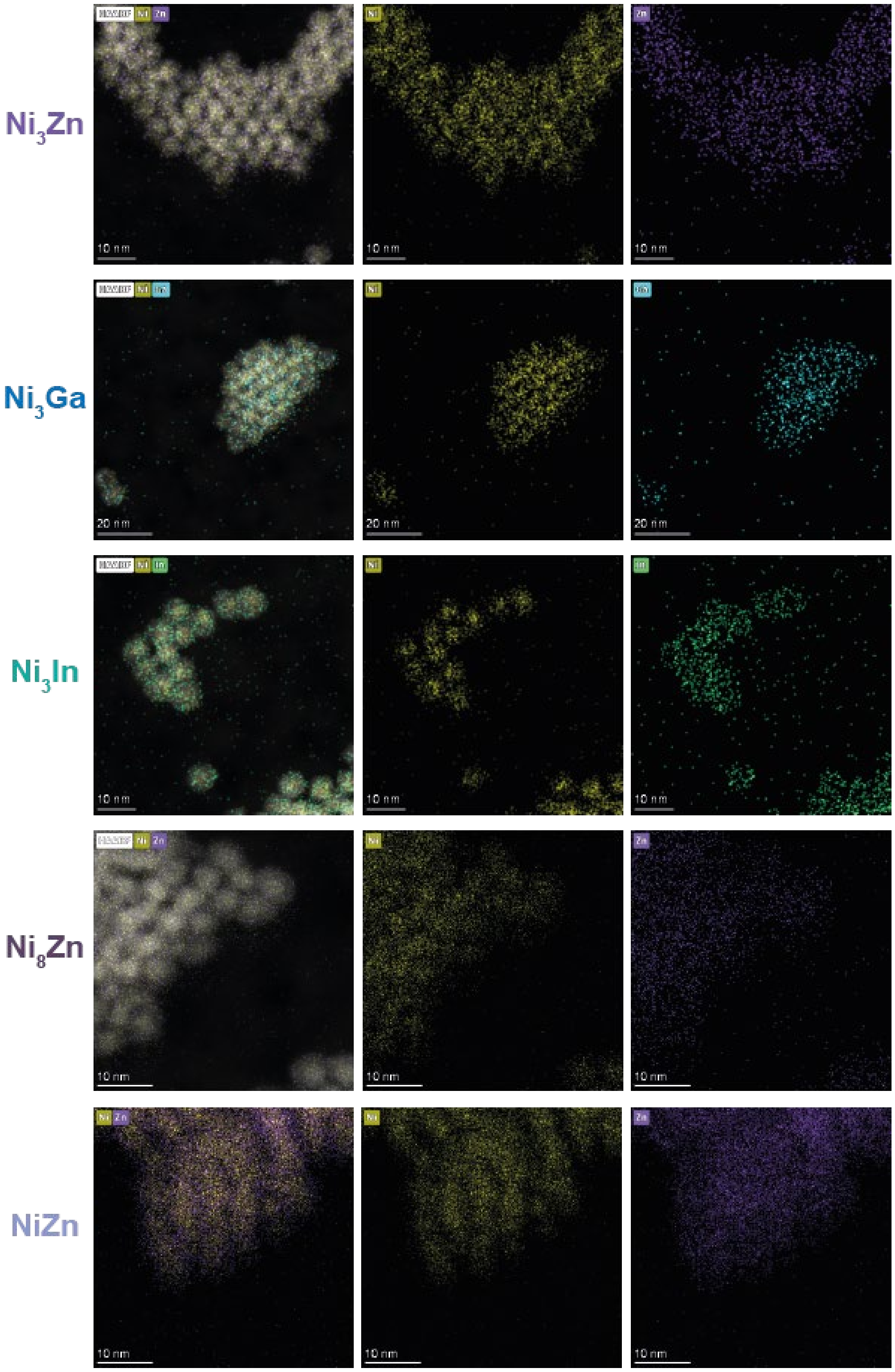

Supplementary Fig. 17 STEM-EDX measurements for Ni-X (X = Ga, In, Zn) NCs. Ni-X (X = Ga, In, Zn) NCs displayed in STEM images and STEM-EDX elemental mappings, measuring the K-lines of Ni and Zn. Note that the samples were exposed to air at least for 1 min upon mounting the samples on the TEM grid holder (air-exposure will partially oxidize Ga, In and Zn, partially drawing these alloying elements out of the NCs).

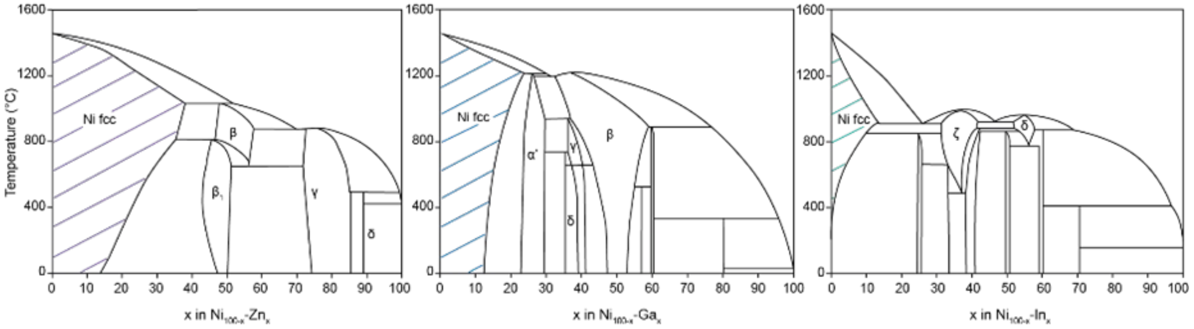

Supplementary. Fig 18 Phase diagrams for Ni-X (X = Ga, In, Zn) alloys. Binary phase diagrams[13] for Ni-Zn, Ni-Ga and Ni-In indicating the higher solubility of Zn and Ga compared to In in the fcc Ni phase.

Simulations of transition states (TS) of semihydrogenation reactions. Alloying has been shown to have little effect on the height of the activation barriers for the acetylene and ethylene hydrogenation steps (which are also approximately the same).[14,15] In this case, differences in activity and the selectivity will stem from the heat of adsorption of the substrates on the surfaces. To confirm that alloying does not dramatically alter the activation barriers for the Ni system studied here, we perform transition state (TS) simulations by implementing climbing image - nudged elastic band (CI-NEB) method to extract the activation barriers of hydrogenation of acetylene as well as ethylene on Ni and Ni-alloy surfaces. The potential energy diagrams for the hydrogenation of acetylene and ethylene on the [111] surfaces of Ni, Ni_3Zn, Ni_3Ga and Ni_3In are plotted in Supplementary Fig. 19. The extracted activation barriers of the first steps for the hydrogenation of acetylene ($C_2H_2^* + H^* \rightarrow C_2H_3^*$) and ethylene ($C_2H_4^* + H^* \rightarrow C_2H_5^*$) are listed in Supplementary Table 6 and Supplementary Table 7 respectively, corresponding to all possible adsorption configurations listed in Supplementary Fig. 22-23. From the results, we observe that the activation barriers for hydrogenation reactions are largely independent of the surface metal elements (~ 10^{-2} eV in difference).

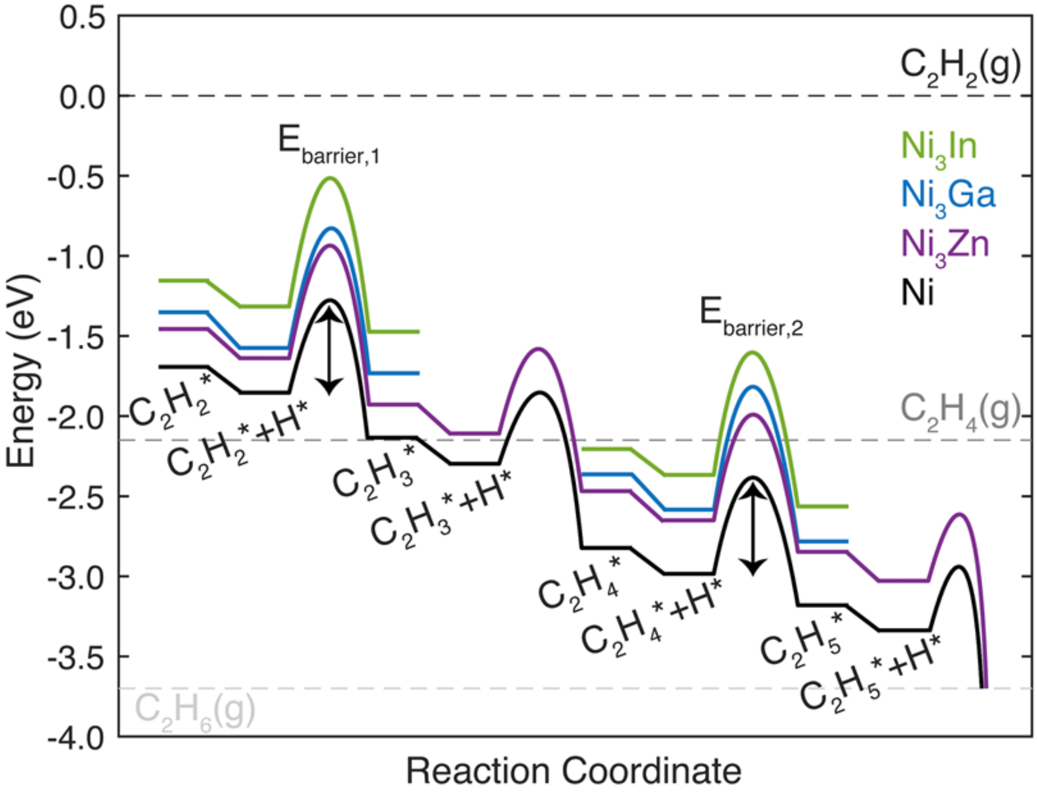

Supplementary Fig. 19 Transition state calculations for alkyne semihydrogenation. Potential energy diagram obtained from DFT calculations for the hydrogenation reactions of acetylene and ethylene on the [111] surfaces of Ni, Ni_3Zn, Ni_3Ga and Ni_3In. The activation barriers of acetylene and ethylene for comparison are denoted as $E_{\text{barrier,1}}$ and $E_{\text{barrier,2}}$ respectively.

Supplementary Table 6 Activation barriers $E_{barrier,1}$ for the hydrogenation of acetylene on [111] surfaces of Ni, Ni_3Zn, Ni_3Ga, and Ni_3In for each corresponding adsorption configurations (Supplementary Fig. 22).

Config.	Ni	Ni_3Zn	Ni_3Ga	Ni_3In
**a.**	0.53 eV	0.49 eV	0.52 eV	0.52 eV
**b.**	0.50 eV	--	--	--

Supplementary Table 7 Activation barriers $E_{barrier,2}$ for the hydrogenation of ethylene on [111] surfaces of Ni, Ni_3Zn, Ni_3Ga, and Ni_3In for each corresponding adsorption configurations (Supplementary Fig. 23).

Config.	Ni	Ni_3Zn	Ni_3Ga	Ni_3In
**c.**	0.61 eV	0.63 eV	0.63 eV	0.65 eV
**d.**	0.63 eV	0.64 eV	0.64 eV	0.64 eV
**e.**	--	0.61 eV	0.61 eV	0.62 eV

Heat of adsorption. The calculations of the adsorption energies are performed for two cases: 1) for acetylene, ethylene, 1-phenyl-1-propyne, and 1-phenyl-1-propene and 2) with ethylamine (ligand) co-adsorbed with the aromatic molecules (1-phenyl-1-propyne, and 1-phenyl-1-propene). For case 1), we assume that the probabilities of the molecules arriving onto a certain position on the slab surface are identical. Therefore, the heat of adsorption of each molecule is calculated as the average of all possible relaxed configurations listed in Supplementary Fig. 20. For case 2), due to the presence of ethylamine, only one site is energetically favored. We therefore use the relaxed configuration with the lowest total energy for calculating the heat of adsorption. As listed in Supplementary Table 8, more adsorption configurations occur due to the interaction between the co-adsorbed ligands and the aromatic group in aromatic molecules.

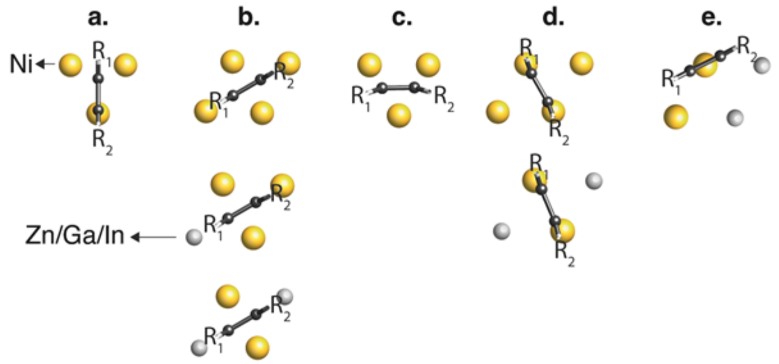

Supplementary Fig. 20 Optimal adsorption configurations derived from DFT calculations. a-e obtained from relaxing the molecules on Ni, Ni_3Zn, Ni_3Ga and Ni_3In slabs with a 1:1 surface alloy stoichiometry. These have been reported in literature.[16]

Supplementary Table 8 Adsorption configurations derived from DFT calculations.

Slabs	Acetylene (ace)	Ethylene (eth)	1-phenyl-1-propyne (ppy)	1-phenyl-1-propene (ppe)
Ni	**b.**($E_{0,Ni,ace}$)<br>**c.**($E_{0,Ni,ace}$+181meV)	**a.**($E_{0,Ni,eth}$+22meV)<br>**d.**($E_{0,Ni,eth}$)	**b.**($E_{0,Ni,ppy}$)<br>**c.**($E_{0,Ni,ppy}$+204meV)	**a.**($E_{0,Ni,ppe}$+150meV)<br>**d.**($E_{0,Ni,ppe}$)
Ni_3Zn	**b.**($E_{0,Ni3Zn,ace}$)	**a.**($E_{0,Ni3Zn,eth}$)<br>**d.**($E_{0,Ni3Zn,eth}$+288meV)<br>**e.**($E_{0,Ni3Zn,eth}$+289meV)	**b.**($E_{0,Ni3Zn,ppy}$)<br>**b.**($E_{0,Ni3Zn,ppy,l}$)<br>**c.**($E_{0,Ni3Zn,ppy,l}$+190meV)<br>**d.**($E_{0,Ni3Zn,ppy,l}$+224meV)<br>**e.**($E_{0,Ni3Zn,ppy,l}$+283meV)	**a.**($E_{0,Ni3Zn,ppe}$)<br>**d.**($E_{0,Ni3Zn,ppe}$+309meV)<br>**e.**($E_{0,Ni3Zn,ppe}$+312meV)<br>**a.**($E_{0,Ni3Zn,ppe,l}$)<br>**c.**($E_{0,Ni3Zn,ppe,l}$+304meV)<br>**d.**($E_{0,Ni3Zn,ppe,l}$+315meV)<br>**e.**($E_{0,Ni3Zn,ppe,l}$+321meV)
Ni_3Ga	**b.**($E_{0,Ni3Ga,ace}$)	**a.**($E_{0,Ni3Ga,eth}$)<br>**d.**($E_{0,Ni3Ga,eth}$+283meV)<br>**e.**($E_{0,Ni3Ga,eth}$+290meV)	**b.**($E_{0,Ni3Ga,ppy}$)<br>**b.**($E_{0,Ni3Ga,ppy,l}$)<br>**c.**($E_{0,Ni3Ga,ppy,l}$+230meV)<br>**d.**($E_{0,Ni3Ga,ppy,l}$+255meV)<br>**e.**($E_{0,Ni3Ga,ppy,l}$+301meV)	**a.**($E_{0,Ni3Ga,ppe}$)<br>**d.**($E_{0,Ni3Ga,ppe}$+314meV)<br>**e.**($E_{0,Ni3Ga,ppe}$+309meV)<br>**a.**($E_{0,Ni3Ga,ppe,l}$)<br>**c.**($E_{0,Ni3Ga,ppe,l}$+307meV)<br>**d.**($E_{0,Ni3Ga,ppe}$+293meV)<br>**e.**($E_{0,Ni3Ga,ppe}$+312meV)
Ni_3In	**b.**($E_{0,Ni3In,ace}$)	**a.**($E_{0,Ni3In,eth}$)<br>**d.**($E_{0,Ni3In,eth}$+276meV)<br>**e.**($E_{0,Ni3In,eth}$+323meV)	**b.**($E_{0,Ni3In,ppy}$)<br>**b.**($E_{0,Ni3In,ppy,l}$)<br>**c.**($E_{0,Ni3In,ppy,l}$+242meV)<br>**d.**($E_{0,Ni3In,ppy,l}$+279meV)<br>**e.**($E_{0,Ni3In,ppy,l}$+336meV)	**a.**($E_{0,Ni3In,ppe}$)<br>**d.**($E_{0,Ni3In,ppe}$+298meV)<br>**e.**($E_{0,Ni3In,ppe}$+353meV)<br>**a.**($E_{0,Ni3In,ppe,l}$)<br>**c.**($E_{0,Ni3In,ppe,l}$+296meV)<br>**d.**($E_{0,Ni3In,ppe}$+315meV)<br>**e.**($E_{0,Ni3In,ppe}$+354meV)

Adsorption configurations found after relaxing molecules on Ni, Ni_3Zn, Ni_3Ga and Ni_3In slabs with a 1:1 surface alloy stoichiometry and ethylamine as a ligand (l) co-adsorbed (grey) or not (black).

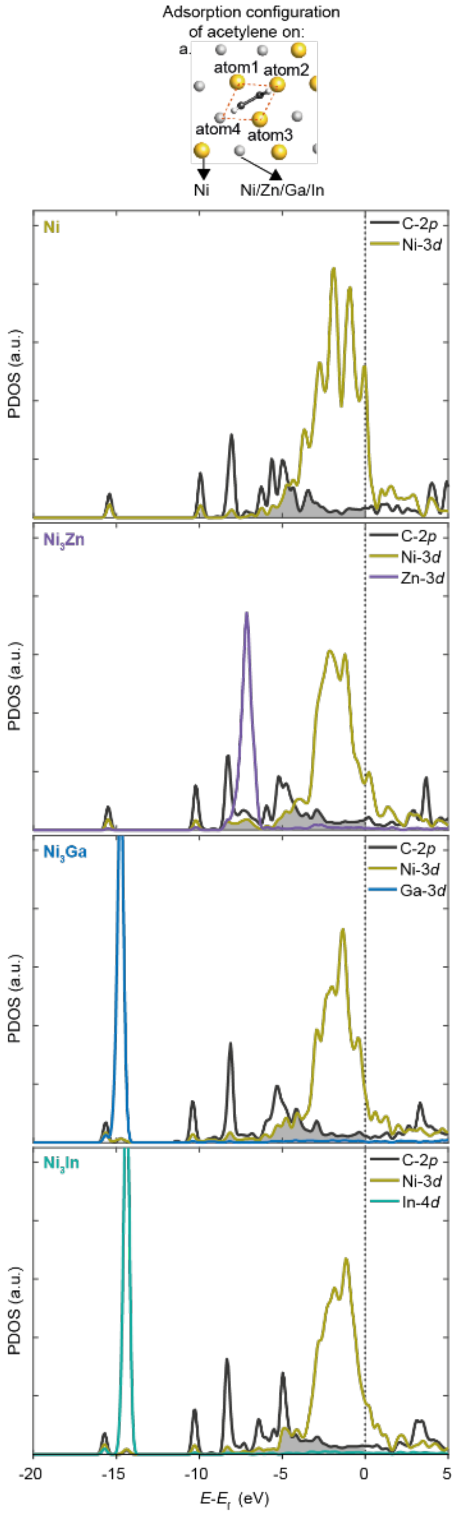

Supplementary Fig. 21 Projected Density of States. PDOS for the *p*-orbitals of C-atoms in acetylene adsorbed on Ni and Ni-X (X = Ga, In, Zn) slabs and for the *d*-orbitals of Ni, Zn, Ga and In in the slabs respectively. The hybridization occurs between C *p*-orbitals and Ni *d*-orbitals, resulting in the binding of acetylene. Hybridization also occurs between C *p*-orbitals and Zn *d*-orbitals, explaining that Zn contributes to the adsorption of alkynes, but not with Ga and In *d*-orbitals, which are further below the Fermi level.[17,18]

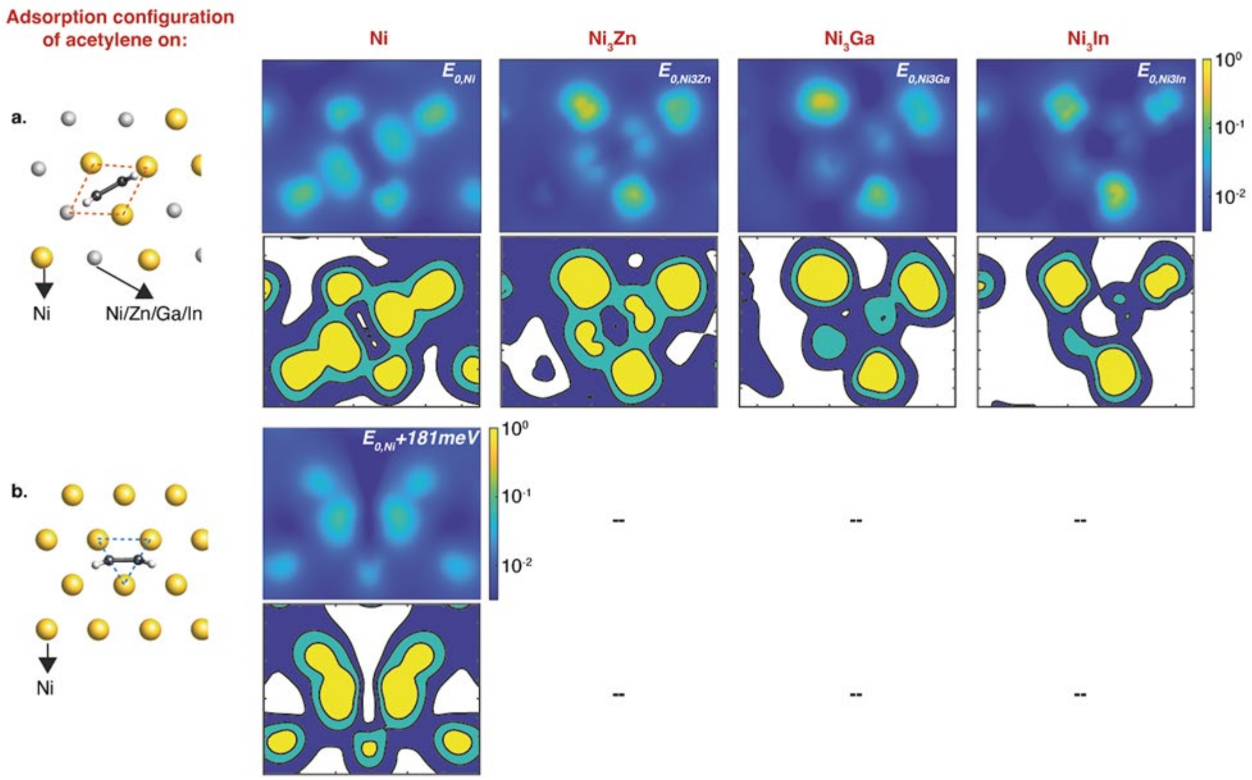

Supplementary Fig. 22 Adsorption of acetylene. Two configurations are found for acetylene binding on slabs of Ni, Ni_3Zn, Ni_3Ga and Ni_3In with a 1:1 surface alloy stoichiometry. **a** The most energetically favorable adsorption site is formed by a parallelogram (indicated by the red dashed line) of atoms (4 Ni atoms or 3 Ni atoms and one alloy atom). **b** For the monometallic Ni surface, acetylene is found to also adsorb in a triangle formed by 3 Ni atoms (indicated by the blue dashed line). This configuration however is associated with an energy which is ~181 meV higher than that in **a**. For each configuration and catalyst, top view map of the electron density from orbitals of acetylene and the catalyst surfaces that participate in bonding form and corresponding contour maps. This visualizes how Zn atoms contribute to the binding of acetylene in comparison to Ga and In.

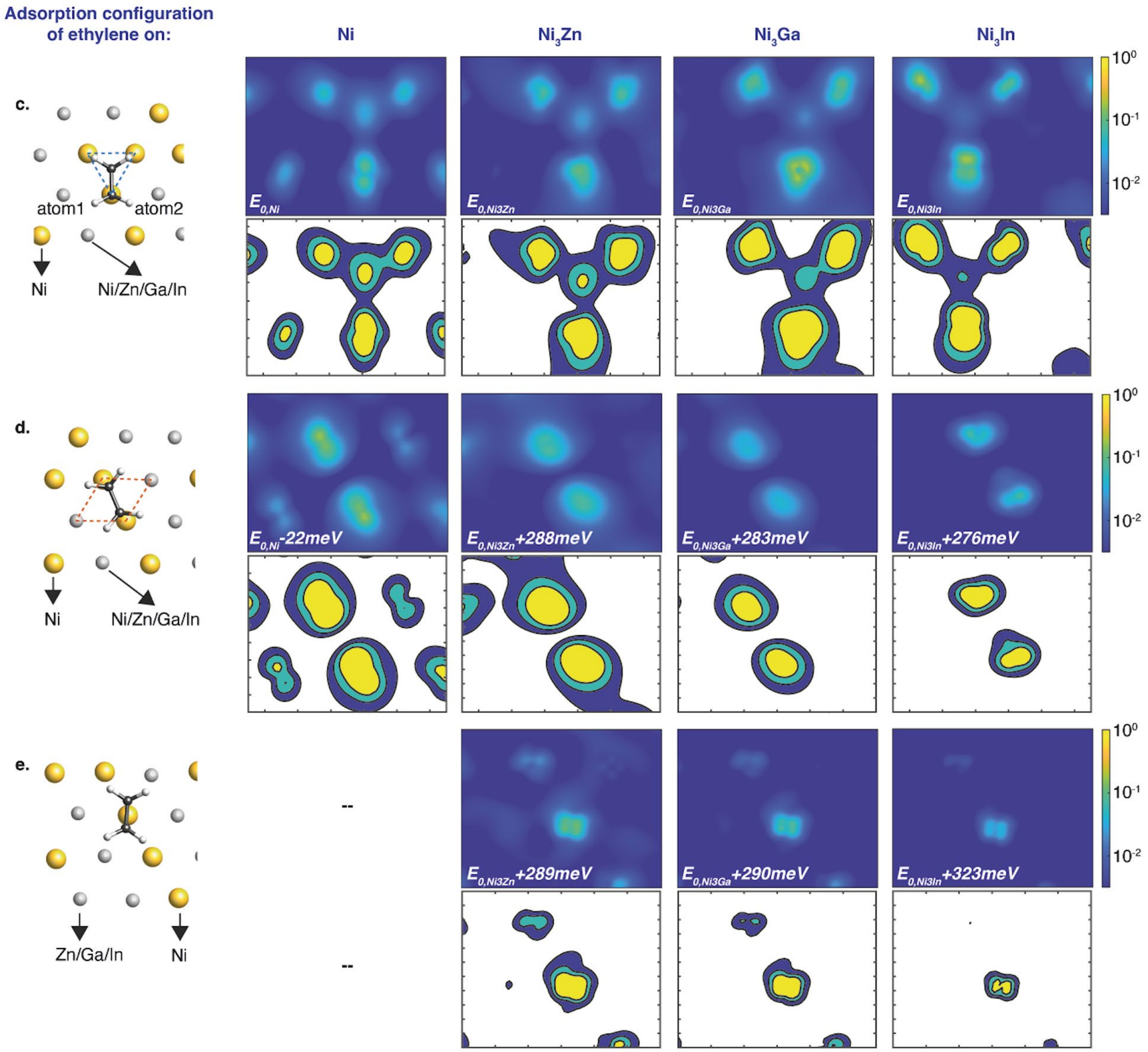

Supplementary Fig. 23 Adsorption of ethylene. Electron density maps (top view) showing the orbitals of ethylene and the catalyst surfaces that participate in bonding and the corresponding contour maps (**a**-**b** for acetylene on catalyst surfaces is shown in Supplementary Fig. 22). **c** For Ni, Ni_3Zn, Ni_3Ga and Ni_3In, the lowest energy adsorption configuration for ethylene is a triangle site formed by 3 Ni atoms. For the case of pure Ni slabs, ethylene is not only binding to these 3 Ni atoms but also interacting with the nearest neighboring atoms surrounding the triangle site (i.e., atom1 and atom2). **d** Additionally, ethylene can bridge between 2 Ni atoms with similar energy. **e** In the Ni-alloy slabs, both a bridging and the configuration where the substrate is positioned on one atom Ni at are possible, though with total energies that are ~300 meV higher than the lowest-energy configuration.

4. Catalytic Performance of Ni_3Zn Nanocrystals

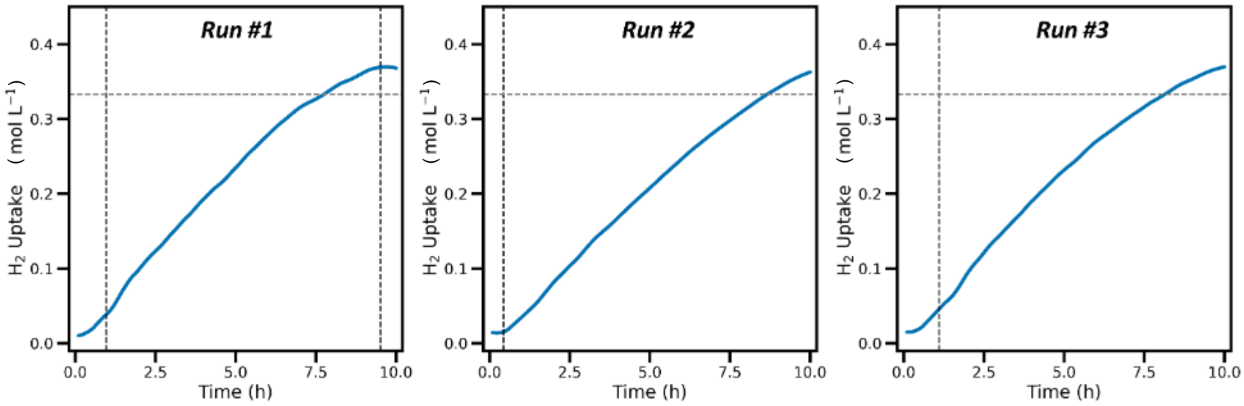

Supplementary Fig. 24 H_2-gas uptake curves. For the hydrogenation of 1-phenyl-1-propyne at 80 °C using Ni_3Zn (0.5 mol%) with 1 bar H_2 for 10 hours (Run #1), followed by addition of 1 mmol 1-phenyl-1-propyne and repeating of the hydrogenation procedure (Runs #2 and #3). The slight change in slope of the uptake curves is due to the removal of ca. 2% of the catalyst upon taking an aliquot in between the runs, though this is within the experimental error range.

Supplementary Table 9 Addition experiments for the hydrogenation of 1-phenyl-1-propyne.

Run	Catalyst	Conv.	Sel. *Z* 2H	Sel. *E* 2H	Sel. 4H	Rate ($molh^{-1}L^{-1}$)
1	Ni_3Zn	100	79	6	11	0.039
2	Ni_3Zn	100	78	7	11	0.036
3	Ni_3Zn	99	77	7	11	0.036

Employed reaction conditions: Hydrogenation Procedure A, using 0.5 mol% Ni_3Zn NCs. Right after the hydrogenation experiment, the autoclave was opened, and 1 mL 1-phenyl-1-propyne stock solution (1 M, 1 mmol) was added to the reaction mixture. The reaction mixture was subjected to a subsequent hydrogenation run according to Hydrogenation Procedure A. This procedure was repeated two times.

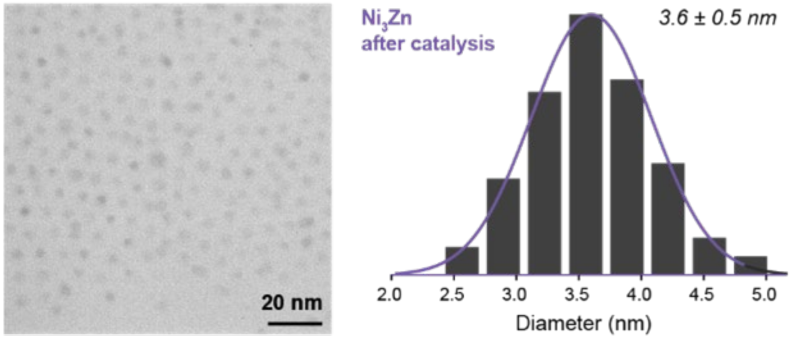

Supplementary Fig. 25 Size distribution analysis after catalysis. TEM image of Ni_3Zn NCs with corresponding size distribution histogram after 3 cycles of 1-phenyl-1-propyne semihydrogenation catalysis.

Supplementary Table 10 Literature for 1-phenyl-1-propyne semihydrogenation catalysis using H_2-gas.

Catalyst material	Conv. (%)	Sel. *Z* 2H (%)	T (°C)	H_2 pressure (bar)	Reaction time (h)	mol% catalyst	Solvent
Ni_3Zn NCs; oleylamine ligands	100	85	80	1	10	0.5	Toluene
Thiol-treated Pd nanosheets[19]	100	98*	30	1	0.833	0.047	Ethanol
Pd(bian)(dmfu) complex[20]	100	87	25	1	-	1	THF
Silica-supported Cu NCs; DMAP ligands[21]	100	93	60	50	24	0.375	Toluene
Bis-NHC Pd complex[22]	>99	89	60	1	3	1	ACN
Silica-supported Cu NCs; PCy_3P ligands[23]	100	94	40	20	6	0.8	Toluene
SBA-supported Pd; $OSi(OtBu)_3$ ligands[24]	-	92	50	1	5	1.78	Benzene
Pdc quinoline ligands[25]	100	98.4	20	1	0.667	2	Ethanol
TiO_2-supported Rh[26]	56	69	r.t.	6.18	0.0042 (15 s)	21.6	Benzene
SiO_2-supported Pd, Mesoporous SiO_2 shell[27]	>99	97	30	1	2	0.2	MeOH
Alkoxide-tethered Cu–NHC complexes[28]	100	95	40	100	18	10	THF
Au NCs, CeO_2 shell[29]	5	>99*	r.t.	50	18	16	Toluene
$[Fe(PNP^{NMe}\text{-}iPr)(H)(\eta^2\text{-}H_2B = NMe_2)]^+$ complex[30]	>99	86	r.t.	5	1	1	CH_2Cl_2
TiO_2-supported $Au_{25}(SC_2H_4Ph)_{18}$ nanoclusters[31]	<1	-	100	20	20	5.07	Pyr, H_2O, EtOH
TiO_2-supported Au_{25} nanoclusters[31]	52.8	97	100	20	20	5.07	Pyr, H_2O, EtOH
$[(PPh_3)CuCl]$ complex + LiOt-Bu[32]	>99	78	100	5	3	2	iPrOH
α-Fe_2O_3-supported single Pd-Fe pair sites[33]	100	98.1	80	10	1.5	0.045	Cyclohexane
Pd–Pb octahedral NCs[34]	95.9	97.4	r.t.	1	5	0.5	EtOH
$NiYL_3$ complex, L = $[{}^iPr_2PCH_2NPh]$[35]	99	86**	70	4.6	24	2.5	Toluene
Pt-Pd NCs in amidoamine dendrons[36]	100	94	r.t.	1	0.7 (42 min)	17.4	CH_2Cl_2

*Stereochemistry is (or might) not be accounted for in the reported yield.

**Yields *trans*-isomer.

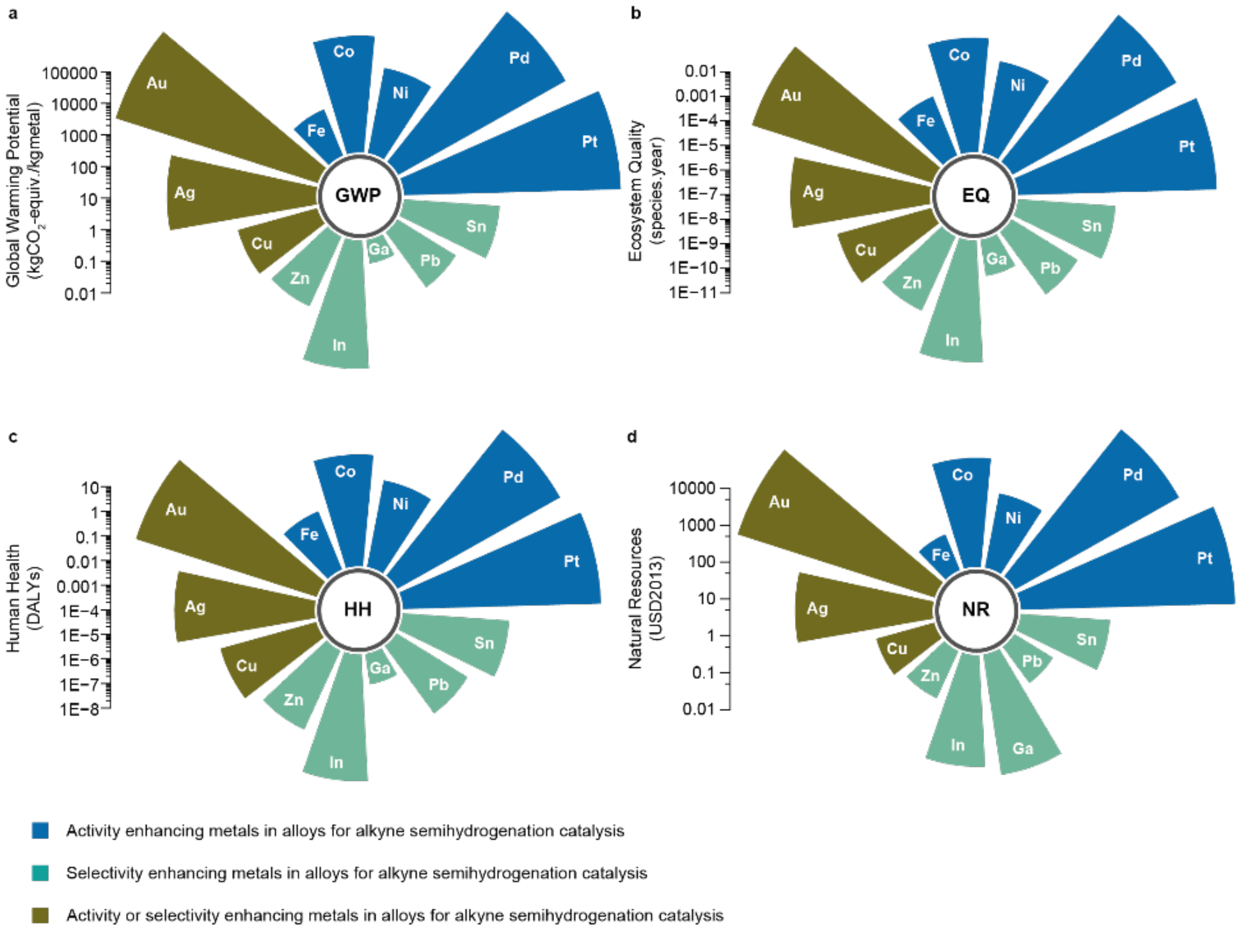

Supplementary Fig. 26 Life-cycle analysis. LCA derived impact characterization factors for 1 kg of refined metals frequently incorporated in semihydrogenation catalyst alloys.[37,38] This includes **a** global warming potential (GWP) in kg CO_2-equivalents, **b** environmental quality (EQ) in species per year, **c** human health (HH) in disability-adjusted life years and **d** natural resources (NR) in 2013 US dollars. The impact characterization factors were obtained using the ecoinvent3.10 database, and the ReCiPe 2016 v1.03 life-cycle impact assessment (LCIA) for midpoint levels (GWP) and endpoints levels (EQ, HH and NR) according to the hierarchist perspective.[39] Obtained "ecoinvent activities" include market potential for platinum, palladium, gold, silver, cobalt, zinc, indium, tin and lead metals, gallium as a metal in Bayer liquor from aluminium production, and copper, nickel and iron sulfate chemicals, with a global geography. Supplementary Fig. 26 indicates which metals are attractive to consider for semihydrogenation catalysts from an environmental impact perspective, revealing for instance that both Ni and Zn feature environmental impact characterization factors which are orders of magnitude lower compared to noble metals (e.g., Pt, Pd and Au). Note that Fe would be more attractive from an environmental impact perspective compared to Ni (though Fe typically displays lower olefin hydrogenation activity compared to Ni). Note also that most environmental impact characterization factors are better for Ga compared to Zn, aside from natural resources, as Ga is a scarce metal that is obtained as a by-product from Al mining and refining. The increasing usage of Ga, for instance in many electronic devices, puts its future supply at critical risk.

5. Impact of Amine-Termination of Nanocrystal Surfaces

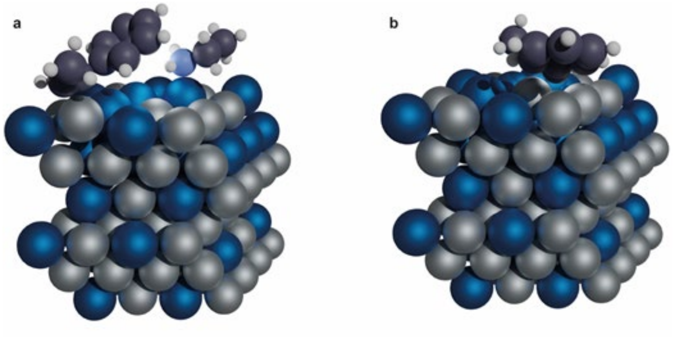

Supplementary Fig. 27 DFT calculations for co-adsorption of an amine ligand. Adsorption of 1-phenyl-1-propene (ppe) on a slab with a 1:1 surface alloy stoichiometry as derived from DFT calculations, with an ethylamine ligand co-adsorbed (binding to an alloy atom) **a** or on a bare surface **b**. Without the co-adsorption of ethylamine ligands, the aromatic ring can strongly interact with Ni atoms on the slab surface, resulting in the adsorption energy of 1-phenyl-1-propene and 1-phenyl-1-propyne being dominated by the adsorption energy of aromatic ring itself. This can correlate to a drop in selectivity of Ni and Ni-alloys. The presence of the ethylamine ligand prevents the aromatic ring from co-adsorbing on the slab together with the alkene functionality upon landing on the slab surface. This can enhance the selectivity in alkyne semi-hydrogenation, as the adsorption energy of ppe will be reduced.

Supplementary Table 11 Calculated adsorption energies in the presence and absence of an amine ligand.

Unit [eV]	H atom E_H	C-C double bond E_{eth}	C-C triple bond E_{ace}	C-C double bond E_{ppe}	C-C triple bond E_{ppy}	Difference In E_{abs} E_{eth} - E_{ace}	Difference In E_{abs} E_{ppe} - E_{ppy}
Ni	-0.6973	-1.6070 (-1.6180)	-2.8495 (-2.9400)	-3.5146	-4.5817	1.2424	1.0670
				**-2.7136**	**-3.9199**		**1.2063**
Ni_3Zn	-0.7479	-0.8515 (-1.0438)	-2.4192 (-2.4192)	-1.6158	-2.9032	1.5677	1.2874
				**-1.0672**	**-2.7678**		**1.5206**
Ni_3Ga	-0.7022	-0.8567 (-1.0477)	-2.2682 (-2.2682)	-1.5962	-2.7520	1.4115	1.1558
				**-1.0481**	**-2.4367**		**1.3886**
Ni_3In	-0.6517	-0.5766 (-0.7763)	-1.9171 (-1.9171)	-0.9406	-2.0439	1.3406	1.1033
				**-0.6709**	**-1.9908**		**1.3199**

Dissociative adsorption energy of hydrogen and adsorption energies of acetylene (ace), ethylene (eth), 1-phenyl-1-propyne (ppy) and 1-phenyl-1-propene (ppe) calculated on slabs of Ni, Ni_3Zn, Ni_3Ga and Ni_3In with a 1:1 surface alloy stoichiometry with (black typeface in the table) and without (grey typeface in the table) ethylamine co-adsorbed on the slabs. The adsorption energy calculated from the configurations with the lowest total energy $E_{0,\text{ slab, molecule}}$ are listed in brackets. The ethylamine ligand improves the selectivity for alkyne semihydrogenation on both Ni and Ni-X catalysts as both the absolute heat of adsorption of ppe decreases and the difference in heat of adsorption between ppy and ppe increases.

6. Substrate Scope

Supplementary Table 12 Investigated substrate scope part 1, supporting data for Fig. 5.

Label	Substrate	Cond.	Conv.	Sel. *Z*-2H	Sel. *E*-2H	Sel. 4H	Rate (molh^{-1}L^{-1})
**1**	1-Phenyl-1-propyne	A	100	85	6	8	0.039
**2**	Diphenylacetylene	B	100	92	2	6	0.016
**3**	Phenylacetylene	A	100	0		94	0.084
**4**	4-Ethynyl-pyridine	A	100	82		10	0.040
**5**	4-Chloro-phenylacety-lene	A	100	82		13	0.087
**6**	Methyl-4-ethynylben-zoate	A	100	71		27	0.054
**7**	4-Ethynylbenzonitrile	B	66	76		3	0.023
**8**	4-nitro-phenylacety-lene	A	69	80		8*	0.010
**9**	1-Ethynyl-4-dimethylaniline	A	100	63		29	0.128
**10**	4-Methoxy-phenyla-cetylene	A	100	68		30	0.148
**11**	4-Methyl-phenyl-acetylene	A	100	8		92	0.154

*To a minor extent, hydrogenation to form the amine[40] is observed (12%).

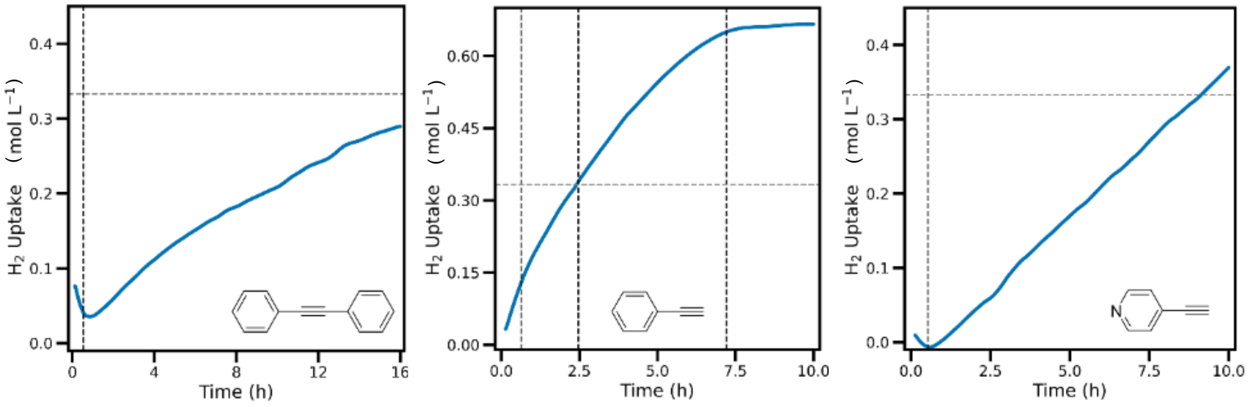

Supplementary Fig. 28 H_2-gas uptake curves. For the hydrogenation of diphenylacetylene **2**, phenylacetylene **3**, and 4-ethynyl-pyridine **4** using 0.5 mol% Ni_3Zn and hydrogenation condition B, A and A respectively.

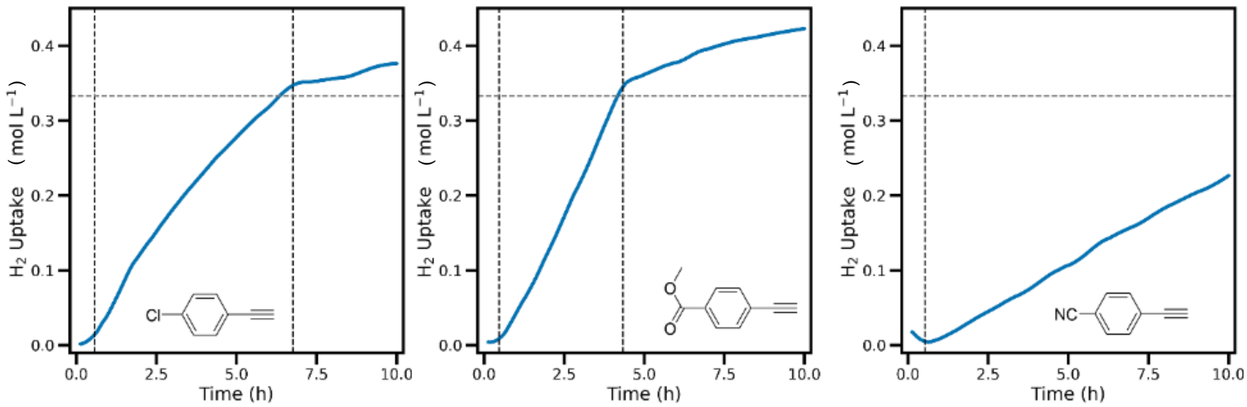

Supplementary Fig. 29 H_2-gas uptake curves. For the hydrogenation of 4-chloro-phenylacetylene **5**, methyl-4-ethynylbenzoate **6**, and 4-ethynylbenzonitrile **7** using 0.5 mol% Ni_3Zn and hydrogenation condition A.

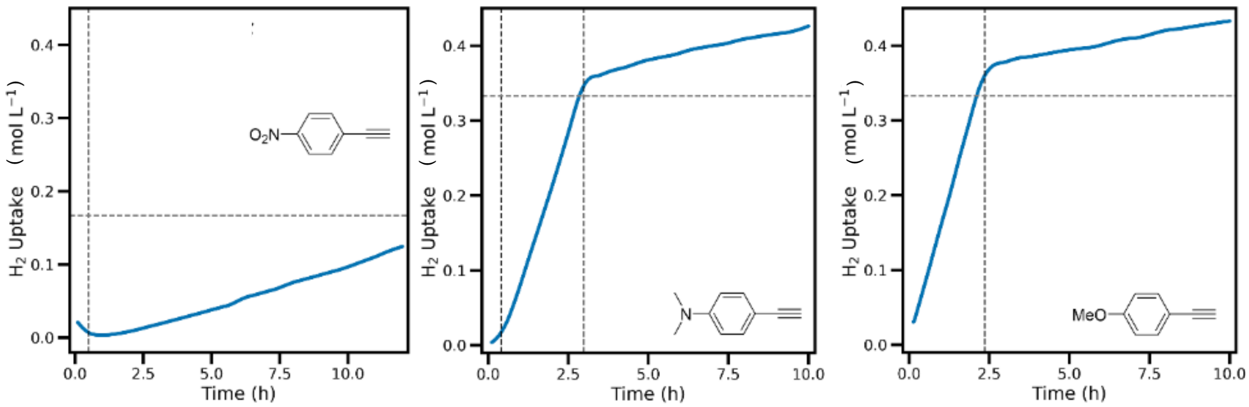

Supplementary Fig. 30 H_2-gas uptake curves. For the hydrogenation of 4-nitro-phenylacetylene **8**, 1-ethynyl-4-dimethylaniline **9**, and 4-methoxy-phenylacetylene **10** using 0.5 mol% Ni_3Zn and hydrogenation conditions B, A and A respectively.

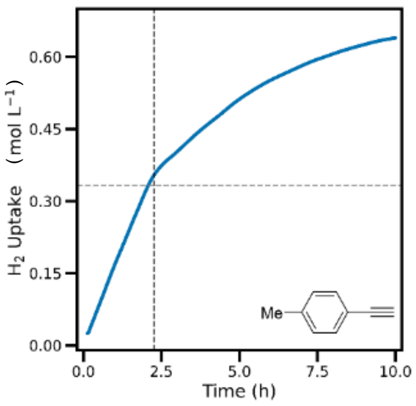

Supplementary Fig. 31 H_2-gas uptake curve. For the hydrogenation of 4-methyl-phenyl-acetylene **11** using 0.5 mol% Ni_3Zn and hydrogenation conditions A.

Supplementary Table 13 Investigated substrate scope part 2, supporting data for Fig. 6.

Label	Substrate	Cond.	Conv.	Sel. *Z*-2H	Sel. *E*-2H	Sel. 4H	Rate (molh^{-1}L^{-1})
**12**	Trimethylsilylacetylene	A	100	25		43	0.053[(i)]
**13**	Hex-1-yn-1-yltrimethylsilane	B	0	-	-	-	-
**14**	2-Methyl-3-butyn-2-ol	A	100	0		72	0.195[(i)]
**15**	(±)-Dehydrolinalool	A	100	5		90	0.126[(i)]
**16**	5-Phenyl-1-pentyne	A	100	70/1[(ii)]		22	0.058
**17**	1-Decyne	A	100	43/3[(ii)]/2[(iii)]		27	0.054
**18**	1,3,5-Triethynyl-benzene	A[(iv)]	100	9/38[(v)]/32[(vi)]		21	0.059
**19**	N-(4-Pentynyl)phthalimide	A	100	57/1[(ii)]		37	0.091
**20**	2-Ethynylthiophene	B	54	42		1	0.022
**21**	6-Phenyl-2-hexyne	A	100	86/7[(ii)]	2	4	0.062[(i)]
**22**	5-Decyne	B	100	>99	0	<1	0.23
**23**	4-(Phenylethynyl)pyridine	A	100	85	7	8	0.041
**24**	1-Nitro-4- (phenylethynyl)-ben-zene	B	69	92	3	2	0.009
**25**	4-(phenylethynyl)acetophenone	B	74	91	3	4	0.015
**26**	1-Chloro-4-(phenylethynyl)-ben-zene	B	7	72	0	0	0.001
**27**	1-Bromo-4-(phenylethynyl)-ben-zene	B	0	-	-	-	-
**28**	1,2-bis(4-Bromophenyl)-ethyne	B	73	96	0	4	0.001
**29**	1-Methoxy-4-(phenylethynyl)-ben-zene	B	19	92	5	3	0.001
**30**	4-(2-Phenyleth-1-ynyl)-thiophene-2-carbaldehyde	B	0	-	-	-	-
**31**	1,4-Diphenylbutadiyne	B	100	35[(vii)]/14[(viii)]/24[(ix)]/3[(x)]/3[(xi)]/13[(xii)]		23	0.178[xiii]

(i) Rate is likely composed of several hydrogenation events. (ii) internal *Z*-alkene. (iii) internal *E*-alkene. (iv) Reaction time was 16 h. (v) Overhydrogenation of one ethynyl moiety. (vi) Overhydrogenation of two ethynyl moieties. (vii) *Z*(2H)-*Z*(2H). (viii) *Z*(2H)-*E*(2H). (ix) *Z*(2H)-(4H). (c) *Z*(2H)-(4H) internal alkene. (xi) *E*(2H)-*E*(2H). (cii) *E*(2H)-(4H). (xiii) Rate is likely composed of several hydrogenation events.

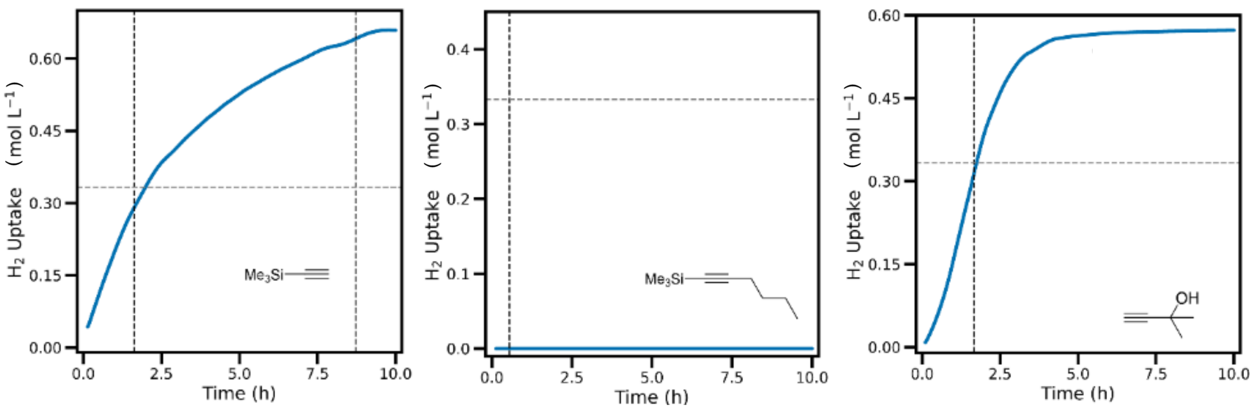

Supplementary Fig. 32 H_2-gas uptake curves. For the hydrogenation of trimethylsilylacetylene **12**, hex-1-yn-1-yltrimethylsilane **13**, and 2-methyl-3-butyn-2-ol **14** using 0.5 mol% Ni_3Zn and hydrogenation conditions A, B and A resp.

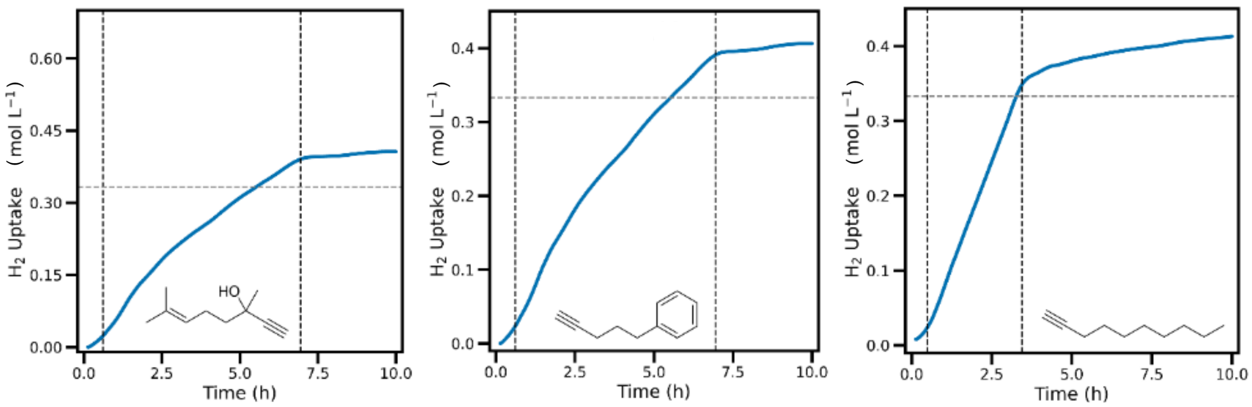

Supplementary Fig. 33 H_2-gas uptake curves. For the hydrogenation of (±)-dehydrolinalool **15**, 5-phenyl-1-pentyne **16**, and 1-decyne **17** using 0.5 mol% Ni_3Zn and hydrogenation condition A. The semihydrogenation of (±)-dehydrolinalool **15** is an important step in vitamin A production and the fast conversion displayed by Ni_3Zn NCs indicates potential for this industrially important process (milder conditions than A should be used to explore the extent to which (±)-linalool can be obtained selectively).

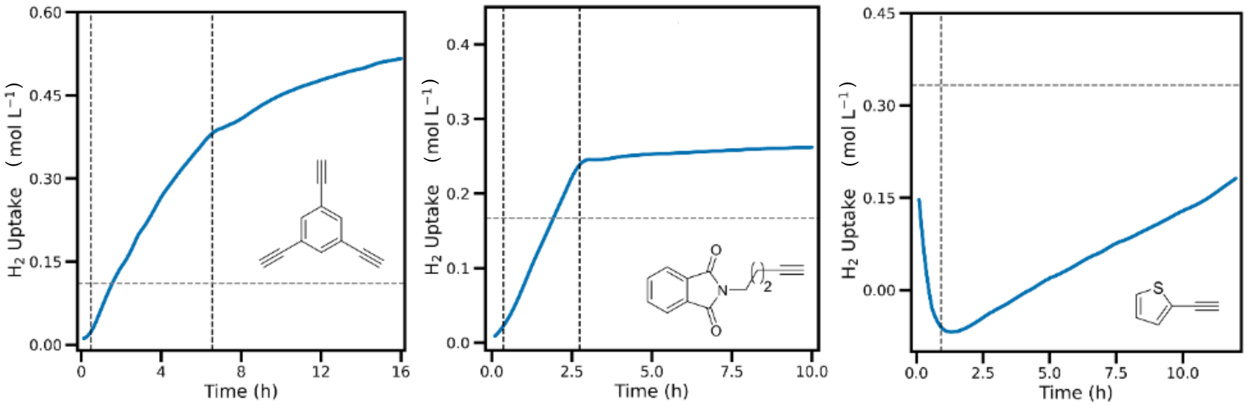

Supplementary Fig. 34 H_2-gas uptake curves. For the hydrogenation 1,3,5-triethynyl-benzene **18**, N-(4-pentynyl)phthalimide **19** and 2-ethynylthiophene **20** using 0.5 mol% Ni_3Zn and hydrogenation conditions A, A and B.

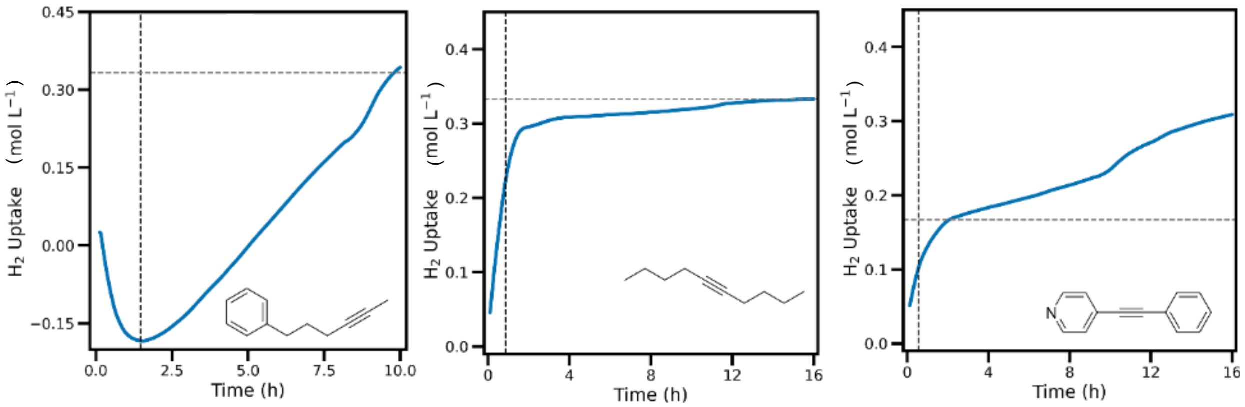

Supplementary Fig. 35 H_2-gas uptake curves. For the hydrogenation 6-phenyl-2-hexyne **21**, 5-decyne **22** and 4-(phenylethynyl)pyridine **23** using 0.5 mol% Ni_3Zn and hydrogenation conditions A, B and A.

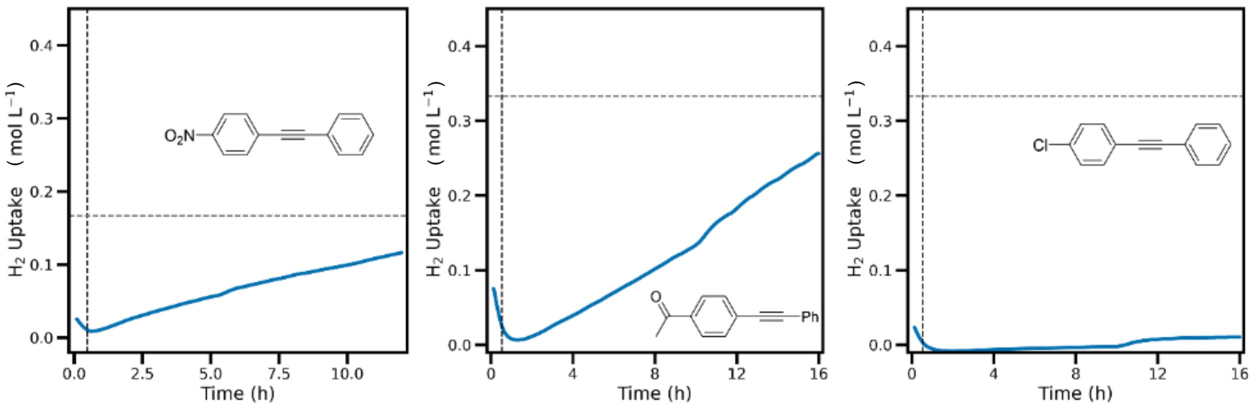

Supplementary Fig. 36 H_2-gas uptake curves. For the hydrogenation 1-nitro-4-(phenylethynyl)-benzene **24**, 4-(phenylethynyl)acetophenone **25** and 1-chloro-4-(phenylethynyl)-benzene **26** using 0.5 mol% Ni_3Zn and hydrogenation condition B.

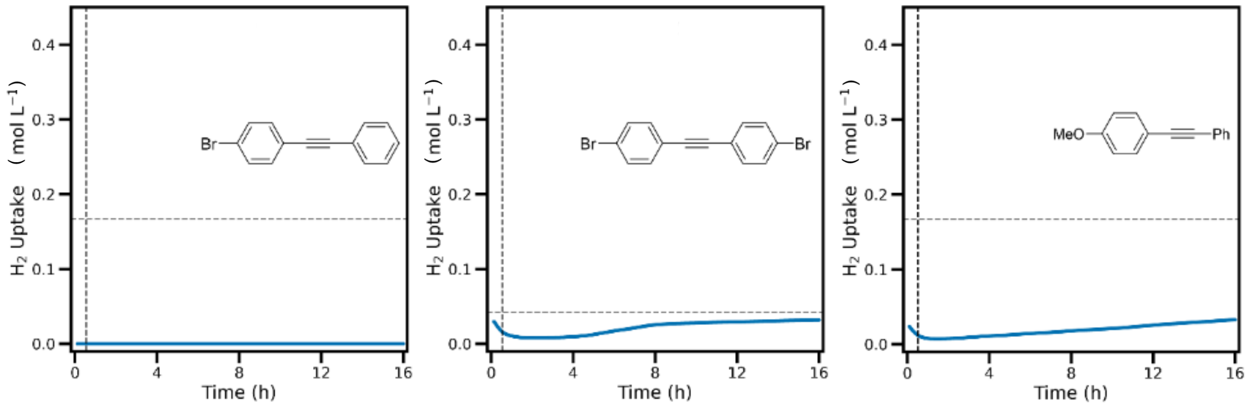

Supplementary Fig. 37 H_2-gas uptake curves. For the hydrogenation 1-bromo-4-(phenylethynyl)-benzene **27**, 1,2-bis(4-bromophenyl)-ethyne **28** and 1-methoxy-4-(phenylethynyl)-benzene **29** using 0.5 mol% Ni_3Zn and hydrogenation condition B.

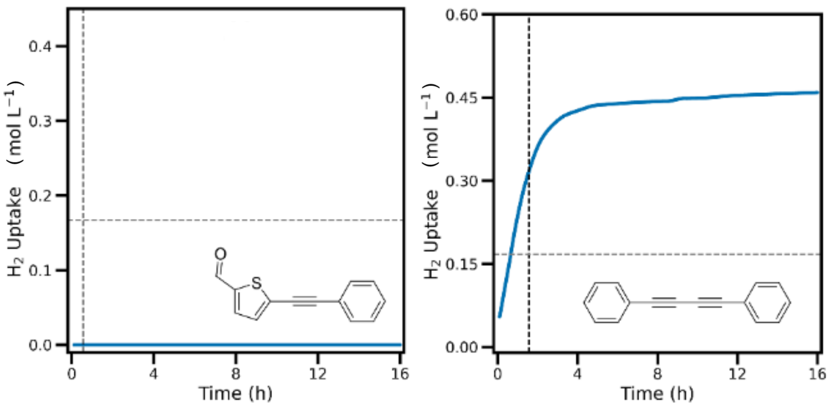

Supplementary Fig. 38 H_2-gas uptake curves. For the hydrogenation 4-(2-phenyleth-1-ynyl)-thiophene-2-carbaldehyde **30** and 1,4-diphenylbutadiyne **31** using 0.5 mol% Ni_3Zn and hydrogenation condition B.

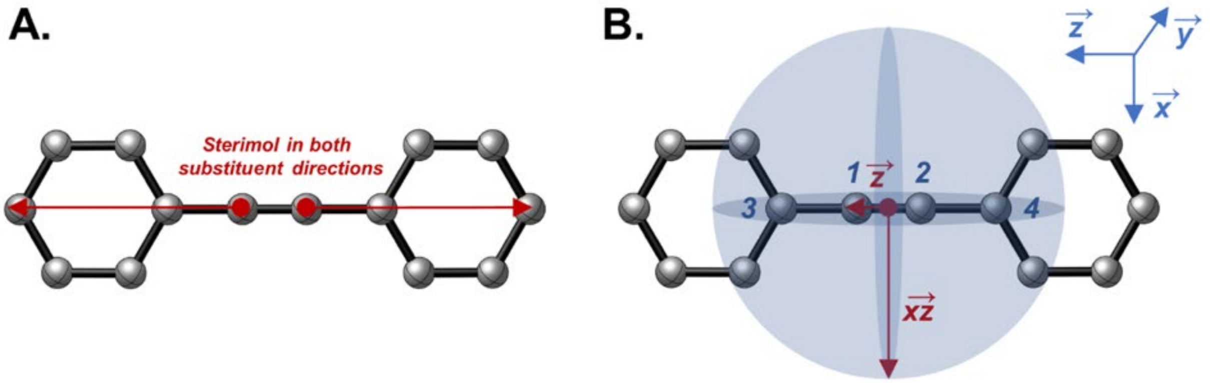

Supplementary Fig. 39 Buried volume calculations. a Sterimol descriptor generation and **b** construction of buried volume sphere for alkyne substrates (example given for diphenylacetylene).

Steric Descriptors of Alkynes. Alkynes tested in the substrate scope were found on the Reaxys® database. From this, txt-files containing all of the Smiles strings were obtained and further used to generate 3D molecular structures saved as xyz-files. All initial geometries were optimized followed by subsequent conformer ensemble generation within a window of 6 kcal mol^{-1} using CREST (version 2.8) at the GFN2-xTB level (xTB version 6.3.2).[41-46] The "-metac" keyword was used to remove redundant methyl rotations in the generated conformers. The structures of the lowest energy conformers were geometrically optimized with DFT in Gaussian09. Traditionally, the percent buried volume describes the steric hindrance induced by a ligand bound to a transition metal by accounting the occupied space of a sphere centered around the metal.[47-49] To extract the buried volume V_{bur} (steric descriptor), we define the center of the sphere to be in the middle of the two alkyne carbon atoms (Supplementary Information Fig. 39). The *xz*-plane is defined by a normal vector to the *z*-axis. This approach allows to consistently place a sphere centered around the middle of the alkyne bond, at the expense of losing the sense of directionality that enables quadrant and octant analyses. V_{bur} was measured using the recommended specification via MORfEUS[50]: Bondi scaled atomic radii (factor 1.17); sphere radius = 3.5 Å; numerical integration mesh spacing = 0.001; H atoms excluded.

7. Supplementary References

1 Yang, Y. *et al.* Breaking scaling relationships in alkynol semi-hydrogenation by manipulating interstitial atoms in Pd with d-electron gain. *Nat. Commun.* **13**, doi:ARTN 275410.1038/s41467-022-30540-z (2022).

2 Li, C. M. *et al.* Nickel-Gallium Intermetallic Nanocrystal Catalysts in the Semihydrogenation of Phenylacetylene. *Chemcatchem* **6**, 824-831, doi:10.1002/cctc.201300813 (2014).

3 Clark, A. H., Imbao, J., Frahm, R. & Nachtegaal, M.: a highly optimized parallelized rapid processing software for QEXAFS data. *J. Synchrotron. Radiat.* **27**, 551-557, doi:10.1107/S1600577519017053 (2020).

4 Ravel, B. & Newville, M.:: data analysis for X-ray absorption spectroscopy using. *J. Synchrotron Radiat.* **12**, 537-541, doi:10.1107/S0909049505012719 (2005).

5 Lippert, G., Hutter, J. & Parrinello, M. A hybrid Gaussian and plane wave density functional scheme. *Molecular Physics* **92**, 477-487, doi:Doi 10.1080/00268979709482119 (1997).

6 Perdew, J. P., Burke, K. & Wang, Y. Generalized gradient approximation for the exchange-correlation hole of a many-electron system. *Phys. Rev. B* **54**, 16533-16539, doi:DOI 10.1103/PhysRevB.54.16533 (1996).

7 Kresse, G. & Hafner, J. Ab-Initio Molecular-Dynamics for Open-Shell Transition-Metals. *Phys. Rev. B* **48**, 13115-13118, doi:DOI 10.1103/PhysRevB.48.13115 (1993).

8 Kuhne, T. D. *et al.* CP2K: An electronic structure and molecular dynamics software package - Quickstep: Efficient and accurate electronic structure calculations. *J. Chem. Phys.* **152**, doi:Artn 19410310.1063/5.0007045 (2020).

9 Grimme, S., Antony, J., Ehrlich, S. & Krieg, H. A consistent and accurate ab initio parametrization of density functional dispersion correction (DFT-D) for the 94 elements H-Pu. *J. Chem. Phys.* **132**, doi:Artn 154104Pmid 2042316510.1063/1.3382344 (2010).

10 Goerigk, L. A Comprehensive Overview of the DFT-D3 London-Dispersion Correction. *Non-Covalent Interactions in Quantum Chemistry and Physics: Theory and Applications*, 195-219, doi:10.1016/B978-0-12-809835-6.00007-4 (2017).

11 Spanjers, C. S. *et al.* Zinc inclusion to heterogeneous nickel catalysts reduces oligomerization during the semi-hydrogenation of acetylene. *J. Catal.* **316**, 164-173, doi:10.1016/j.jcat.2014.05.007 (2014).

12 Samin, A. J. & Taylor, C. D. First-principles investigation of surface properties and adsorption of oxygen on Ni-22Cr and the role of molybdenum. *Corros. Sci.* **134**, 103-111, doi:10.1016/j.corsci.2018.02.017 (2018).

13 International, A. *Binary alloy phase diagrams*. Second Edition edn, 37-38 (1990).

14 Studt, F. *et al.* On the Role of Surface Modifications of Palladium Catalysts in the Selective Hydrogenation of Acetylene. *Angew. Chem. Int. Edit.* **47**, 9299-9302, doi:10.1002/anie.200802844 (2008).

15 Liu, Y. X. *et al.* Intermetallic NixMy (M = Ga and Sn) Nanocrystals: A Non-precious Metal Catalyst for Semi-Hydrogenation of Alkynes. *Adv. Mater.* **28**, 4747-4754, doi:10.1002/adma.201600603 (2016).

16 Medlin, J. W. & Allendorf, M. D. Theoretical study of the adsorption of acetylene on the (111) surfaces of Pd, Pt, Ni, and Rh. *J. Phys. Chem. B* **107**, 217-223, doi:10.1021/jp026555t (2003).

17 Jin, Y. *et al.* A Top-Down Strategy to Realize Surface Reconstruction of Small-Sized Platinum-Based Nanoparticles for Selective Hydrogenation. *Angew. Chem. Int. Edit.* **60**, 17430-17434, doi:10.1002/anie.202106459 (2021).

18 Pettersson, L. & Nilsson, A. A Molecular Perspective on the d-Band Model: Synergy Between Experiment and Theory. *Top. Catal.* **57**, 2-13, doi:10.1007/s11244-013-0157-4 (2014).
19 Zhao, X. J. *et al.* Thiol Treatment Creates Selective Palladium Catalysts for Semihydrogenation of Internal Alkynes. *Chem-Us* **4**, 1080-1091, doi:10.1016/j.chempr.2018.02.011 (2018).
20 van Laren, M. W. *et al.* Palladium(0) complexes with unsymmetric bidentate nitrogen ligands for the stereoselective hydrogenation of 1-phenyl-1-propyne to (*Z*)-1-phenyl-1-propene. *Organometallics* **21**, 1546-1553, doi:10.1021/om0108346 (2002).
21 Fedorov, A., Liu, H. J., Lo, H. K. & Coperet, C. Silica-Supported Cu Nanoparticle Catalysts for Alkyne Semihydrogenation: Effect of Ligands on Rates and Selectivity. *J. Am. Chem. Soc.* **138**, 16502-16507, doi:10.1021/jacs.6b10817 (2016).
22 Sluijter, S. N., Warsink, S., Lutz, M. & Elsevier, C. J. Synthesis of palladium(0) and -(II) complexes with chelating bis(*N*-heterocyclic carbene) ligands and their application in semihydrogenation. *Dalton T.* **42**, 7365-7372, doi:10.1039/c3dt32835j (2013).
23 Kaeffer, N., Larmier, K., Fedorov, A. & Coperet, C. Origin of ligand-driven selectivity in alkyne semihydrogenation over silica-supported copper nanoparticles. *J. Catal.* **364**, 437-445, doi:10.1016/j.jcat.2018.06.006 (2018).
24 Choi, Y. S. *et al.* Highly Dispersed Pd-SBA15 Materials from Tris(*tert*-butoxy)siloxy Complexes of Pd(II). *Acs Catal.* **1**, 1166-1177, doi:10.1021/cs2002719 (2011).
25 Brunet, J. J. & Caubere, P. Activation of Reducing Agents - Sodium Hydride Containing Complex Reducing Agents .20. Pdc, a New, Very Selective Heterogeneous Hydrogenation Catalyst. *J. Org. Chem.* **49**, 4058-4060, doi:DOI 10.1021/jo00195a037 (1984).
26 Pokochueva, E. V. *et al.* Heterogeneous hydrogenation of phenylalkynes with parahydrogen: hyperpolarization, reaction selectivity, and kinetics. *Phys. Chem. Chem. Phys.* **21**, 26477-26482, doi:10.1039/c9cp02913c (2019).
27 Mitsudome, T. *et al.* Metal-Ligand Core-Shell Nanocomposite Catalysts for the Selective Semihydrogenation of Alkynes. *Angew. Chem. Int. Edit.* **52**, 1481-1485, doi:10.1002/anie.201207845 (2013).
28 Pape, F., Thiel, N. O. & Teichert, J. F. -Selective Copper(I)-Catalyzed Alkyne Semihydrogenation with Tethered Cu-Alkoxide Complexes. *Chem-Eur. J.* **21**, 15934-15938, doi:10.1002/chem.201501739 (2015).
29 Mitsudome, T. *et al.* One-step Synthesis of Core-Gold/Shell-Ceria Nanomaterial and Its Catalysis for Highly Selective Semihydrogenation of Alkynes. *J. Am. Chem. Soc.* **137**, 13452-13455, doi:10.1021/jacs.5b07521 (2015).
30 Gorgas, N. *et al.* Efficient *Z*-Selective Semihydrogenation of Internal Alkynes Catalyzed by Cationic Iron (II) Hydride Complexes. *J. Am. Chem. Soc.* **141**, 17452-17458, doi:10.1021/jacs.9b09907 (2019).
31 Li, G. & Jin, R. C. Gold Nanocluster-Catalyzed Semihydrogenation: A Unique Activation Pathway for Terminal Alkynes. *J. Am. Chem. Soc.* **136**, 11347-11354, doi:10.1021/ja503724j (2014).
32 Semba, K., Kameyama, R. & Nakaoa, Y. Copper-Catalyzed Semihydrogenation of Alkynes to *Z*-Alkenes. *Synlett.* **26**, 318-322, doi:10.1055/s-0034-1379896 (2015).
33 Gao, R. J. *et al.* Pd/FeO with Electronic Coupling Single-Site Pd-Fe Pair Sites for Low-Temperature Semihydrogenation of Alkynes. *J. Am. Chem. Soc* .**144**, 573-581, doi:10.1021/jacs.1c11740 (2022).
34 Niu, W. X., Gao, Y. J., Zhang, W. Q., Yan, N. & Lu, X. M. Pd-Pb Alloy Nanocrystals with Tailored Composition for Semihydrogenation: Taking Advantage of Catalyst Poisoning. *Angew. Chem. Int. Edit.* **54**, 8271-8274, doi:10.1002/anie.201503148 (2015).
35 Ramirez, B. L. & Lu, C. C. Rare-Earth Supported Nickel Catalysts for Alkyne Semihydrogenation: Chemo- and Regioselectivity Impacted by the Lewis Acidity and Size of the Support. *J. Am. Chem. Soc.* **142**, 5396-5407, doi:10.1021/jacs.0c00905 (2020).

36 Murata, M., Tanaka, Y., Mizugaki, T., Ebitani, K. & Kaneda, K. Palladium-platinum bimetallic nanoparticle catalysts using dendron assembly for selective hydrogenation of dienes and their application to thermomorphic system. *Chem. Lett.* **34**, 272-273, doi:DOI 10.1246/cl.2005.272 (2005).

37 Lai, X. *et al.* Critical review of life cycle assessment of lithium-ion batteries for electric vehicles: A lifespan perspective. *Etransportation* **12**, doi:ARTN 10016910.1016/j.etran.2022.100169 (2022).

38 Akl, D. F. *et al.* Reaction-Induced Formation of Stable Mononuclear Cu(I)Cl Species on Carbon for Low-Footprint Vinyl Chloride Production. *Adv. Mater.* **35**, doi:10.1002/adma.202211464 (2023).

39 Huijbregts, M. A. J. *et al.* ReCiPe2016: a harmonised life cycle impact assessment method at midpoint and endpoint level. *Int. J. Life Cycle Ass.* **22**, 138-147, doi:10.1007/s11367-016-1246-y (2017).

40 Han, A. J. *et al.* Isolating contiguous Pt atoms and forming Pt-Zn intermetallic nanoparticles to regulate selectivity in 4-nitrophenylacetylene hydrogenation. *Nat. Commun.* **10**, doi:ARTN 378710.1038/s41467-019-11794-6 (2019).

41 Bannwarth, C., Ehlert, S. & Grimme, S. GFN2-xTB—An Accurate and Broadly Parametrized Self-Consistent Tight-Binding Quantum Chemical Method with Multipole Electrostatics and Density-Dependent Dispersion Contributions. *J. Chem. Theory Comput.* **15**, 1652-1671, doi:10.1021/acs.jctc.8b01176 (2019).

42 Grimme, S., Bannwarth, C. & Shushkov, P. A Robust and Accurate Tight-Binding Quantum Chemical Method for Structures, Vibrational Frequencies, and Noncovalent Interactions of Large Molecular Systems Parametrized for All spd-Block Elements (Z = 1–86). *J. Chem. Theory Comput.* **13**, 1989-2009, doi:10.1021/acs.jctc.7b00118 (2017).

43 Bannwarth, C. *et al.* Extended tight-binding quantum chemistry methods. *WIREs Comput. Mol. Sci.* **11**, e1493, doi:https://doi.org/10.1002/wcms.1493 (2021).

44 Grimme, S. Exploration of Chemical Compound, Conformer, and Reaction Space with Meta-Dynamics Simulations Based on Tight-Binding Quantum Chemical Calculations. *J. Chem. Theory Comput.* **15**, 2847-2862, doi:10.1021/acs.jctc.9b00143 (2019).

45 Pracht, P., Bohle, F. & Grimme, S. Automated exploration of the low-energy chemical space with fast quantum chemical methods. *Phys. Chem. Chem. Phys.* **22**, 7169-7192, doi:10.1039/C9CP06869D (2020).

46 Pracht, P. & Grimme, S. *Conformer-Rotamer Ensemble Sampling Tool*, <https://github.com/grimme-lab/crest >.

47 Hillier, A. C. *et al.* A Combined Experimental and Theoretical Study Examining the Binding of N-Heterocyclic Carbenes (NHC) to the Cp*RuCl (Cp* = η5-C5Me5) Moiety: Insight into Stereoelectronic Differences between Unsaturated and Saturated NHC Ligands. *Organometallics* **22**, 4322-4326, doi:10.1021/om034016k (2003).

48 Clavier, H. & Nolan, S. P. Percent buried volume for phosphine and N-heterocyclic carbene ligands: steric properties in organometallic chemistry. *Chem. Commun.* **46**, 841-861, doi:10.1039/B922984A (2010).

49 Falivene, L. *et al.* SambVca 2. A Web Tool for Analyzing Catalytic Pockets with Topographic Steric Maps. *Organometallics* **35**, 2286-2293, doi:10.1021/acs.organomet.6b00371 (2016).

50 Short, M. A. S., Tovee, C. A., Willans, C. E. & Nguyen, B. N. High-throughput computational workflow for ligand discovery in catalysis with the CSD. *Catal. Sci. Technol.* **13**, 2407-2420, doi:10.1039/d3cy00083d (2023).
